# First Insights into Population Structure and Genetic Diversity Versus Host Specificity in Trypanorhynch Tapeworms Using Multiplexed Shotgun Genotyping

**DOI:** 10.1093/gbe/evad190

**Published:** 2023-10-31

**Authors:** Kaylee S Herzog, Jennifer L Hackett, Paul M Hime, Lukas B Klicka, Kirsten Jensen

**Affiliations:** Department of Epidemiology, University of Nebraska Medical Center, Omaha, Nebraska, USA; Biodiversity Institute and Natural History Museum, University of Kansas, Lawrence, Kansas, USA; Department of Ecology & Evolutionary Biology, University of Kansas, Lawrence, Kansas, USA; Genome Sequencing Core, University of Kansas, Lawrence, Kansas, USA; Biodiversity Institute and Natural History Museum, University of Kansas, Lawrence, Kansas, USA; School of Arts and Sciences, Peru State College, Nebraska, USA; Biodiversity Institute and Natural History Museum, University of Kansas, Lawrence, Kansas, USA; Department of Ecology & Evolutionary Biology, University of Kansas, Lawrence, Kansas, USA

**Keywords:** Trypanorhyncha, elasmobranch tapeworms, *Rhinoptericola megacantha*, *Callitetrarhynchus gracilis*, population genomics, RAD-seq

## Abstract

Theory predicts relaxed host specificity and high host vagility should contribute to reduced genetic structure in parasites while strict host specificity and low host vagility should increase genetic structure. Though these predictions are intuitive, they have never been explicitly tested in a population genomic framework. Trypanorhynch tapeworms, which parasitize sharks and rays (elasmobranchs) as definitive hosts, are the only order of elasmobranch tapeworms that exhibit considerable variability in their definitive host specificity. This allows for unique combinations of host use and geographic range, making trypanorhynchs ideal candidates for studying how these traits influence population-level structure and genetic diversity. Multiplexed shotgun genotyping (MSG) data sets were generated to characterize component population structure and infrapopulation diversity for a representative of each trypanorhynch suborder: the ray-hosted *Rhinoptericola megacantha* (Trypanobatoida) and the shark-hosted *Callitetrarhynchus gracilis* (Trypanoselachoida). Adults of *R. megacantha* are more host-specific and less broadly distributed than adults of *C. gracilis*, allowing correlation between these factors and genetic structure. Replicate tapeworm specimens were sequenced from the same host individual, from multiple conspecific hosts within and across geographic regions, and from multiple definitive host species. For *R. megacantha*, population structure coincided with geography rather than host species. For *C. gracilis*, limited population structure was found, suggesting a potential link between degree of host specificity and structure. Conspecific trypanorhynchs from the same host individual were found to be as, or more, genetically divergent from one another as from conspecifics from different host individuals. For both species, high levels of homozygosity and positive F_IS_ values were documented.

**Significance**

While it is intuitive to assume that relaxed host specificity should lead to reduced genetic structure in parasites, this theory has yet to be tested in a population genomic framework. In this study, multiplexed shotgun genotyping data sets were generated for a species each of shark and ray-hosted tapeworms that demonstrate different degrees of host specificity, and population structure was found to coincide with geographic region—rather than definitive host species—in the more host-specific of the two species. These first population genomic data for shark and ray tapeworms provide a primary foundation on which future investigations of parasite population genomics can build.

## Introduction

It has long been suggested that relaxed host specificity and increased host movement should each contribute to reduced genetic structure in parasites, while more host-specific species, as well as those that parasitize less wide-ranging and/or vagile hosts, should exhibit greater degrees of structure (Nadler 1995; Huyse et al. 2005). Though these assumptions are intuitive, they have yet to be tested with modern population genomic data sets for most parasite groups. Overwhelmingly, the group in which the interplay between host associations and population structure has been best studied is the lice. Results based on single and multilocus data sets in various species of avian and mammalian lice have revealed that increased host specificity is positively correlated with increased genetic structure and isolation by distance, and that species with reduced capacities for dispersal are characterized by more structured populations and higher levels of homozygosity and inbreeding (Johnson et al. 2002; Clayton and Johnson 2003; Martinů et al. 2018; Sweet and Johnson 2018; Virrueta Herrera et al. 2022). The results from louse systems clearly support long-standing assumptions and, in part, motivated this investigation of similar patterns in another parasite group.

Perhaps one of the most intriguing groups of parasites from a perspective of population-level structure and diversity is the order Trypanorhyncha, one of eight orders of tapeworms whose members have a complex life-cycle, are trophically transmitted, and exclusively parasitize the digestive system of sharks and rays (i.e., elasmobranchs) as adults (Caira and Jensen 2014). As a group, trypanorhynchs exhibit many unique combinations of host use and geographic range, highlighting them as ideal candidates for studies of how these life history traits may influence population-level structure and genetic diversity. For example, each species of *Hemionchos* is highly host-specific and is known to parasitize only a single species of devil ray (family Mobulidae) (Campbell and Beveridge 2006; Beveridge and Bennett 2022), but individual species of *Prochristianella* have been reported from 39 species of rays representing four orders (Beveridge 1990; Schaeffner and Beveridge 2013, 2014). The geographic ranges exhibited by trypanorhynchs vary somewhat in concert with their varying degrees of host specificity. For example, the host-specific *Prochristianella caribbensis* is known only from the yellow stingray, *Urobatis jamaicensis*, from Jamaica, while *Proemotobothrium linstowi* has been reported from the ocellated eagle ray, *Aetobatus ocellatus* (as *Aetobatis narinari*), the whitespotted wedgefish, *Rhynchobatus djiddensis*, and a species of requiem shark (family Carcharhinidae), collectively from Australia, Singapore, and Sri Lanka (Beveridge and Campbell 2001; Palm 2004).

To date, population genomics in tapeworms has been restricted to a handful of species that infect humans and livestock (e.g., Addy et al. 2017), and a species each infecting freshwater teleosts, clawed frogs, and birds (Brabec et al. 2016; Fraija-Fernández et al. 2021) and has not included tests of the interplay between host specificity and genetic structure. The first insights into the population genomics of trypanorhynch tapeworms are presented here. Two focal species were selected for this investigation, representing each of the two trypanorhynch suborders: *Rhinoptericola megacantha* (family Rhinoptericolidae) in the suborder Trypanobatoida, and *Callitetrarhynchus gracilis* (family Lacistorhynchidae) in the suborder Trypanoselachoida. Both species demonstrate relaxed host specificity, or are euryxenous sensu Caira et al. (2003), but they vary in their degree of euryxeny and in their geographic ranges. Adults of *R*. *megacantha* are known from three species of cownose rays (family Rhinopteridae) and a species of stingray (family Dasyatidae), and they are restricted to the Atlantic Ocean (Herzog and Jensen 2022). *Callitetrarhynchus gracilis* is comparably less host-specific and more broadly distributed. Prior to this study, adults of *C. gracilis* had been reported from 20 species of carcharhiniform sharks representing eight genera and three families (i.e., Carcharhinidae, Sphyrnidae, and Triakidae) (Nakajima and Egusa 1972a, 1972b, 1972c, 1973; Heinz and Dailey 1974; Watson and Thorson 1976; São Clemente and Gomes 1989; Palm 1995; Palm and Overstreet 2000; Olson et al. 2001; Palm 2004; Owens 2008; Haseli et al. 2010; Olson et al. 2010; Méndez and Dorantes González 2013; Schaeffner and Beveridge 2014; Mhaisen et al. 2018), and from a species each of ginglymostomatid wobbegong (Beveridge and Campbell 1998) and dasyatid stingray (Palm 2004). These reports include localities across the world's tropical and temperate oceans, and even include a report from the bull shark, *Carcharhinus leucas*, from freshwater river systems in Costa Rica (Watson and Thorson 1976).

The goal of this investigation was to leverage next generation sequencing methods and previous global collections of trypanorhynchs from elasmobranchs with an emphasis on broad and deep sampling to address the following questions: 1) Is population-level genetic diversity in trypanorhynch tapeworms that demonstrate relaxed host specificity structured by definitive host species or geography? 2) Does the degree of host specify correlate with observed patterns of population structure? 3) Are conspecific trypanorhynchs more genetically similar within a host individual than between host individuals?

## Results

### Sampling Across Known Host Species and Geographic Localities

**Fig. 1.**
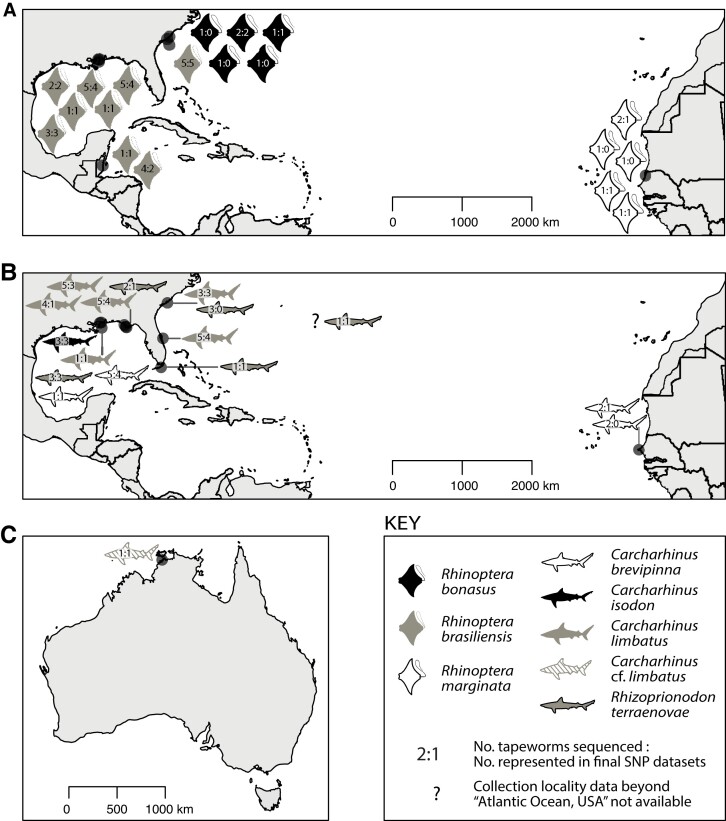
Maps of sampling localities indicating the number of individuals of each host species sampled per locality and the number of tapeworms sequenced per host individual for *Rhinoptericola megacantha* (*A*) and *Callitetrarhynchus gracilis* (*B–C*).

For *R. megacantha*, 39 specimens were sequenced. They were collected from specimens of *Rhinoptera bonasus* (South Carolina), *Rhinoptera brasiliensis* (Belize, Gulf of Mexico, and South Carolina), and *Rhinoptera marginata* (Senegal) (see fig. 1, supplementary Tables S1 and S2, Supplementary Material online). Specimens from the only other species of elasmobranch host from which *R. megacantha* has been reported (i.e., the dasyatid *Hypanus say*), and from three additional geographic localities from which it is known (i.e., the Chesapeake Bay, the Gulf of Venezuela, and Brazil) were unavailable (see supplementary Tables S3 and S4, Supplementary Material online). None of these 39 specimens represents a novel record of host species or geographic locality for *R. megacantha* (see supplementary Table S3, Supplementary Material online).

For *C. gracilis*, 47 specimens were sequenced. They were collected from specimens of *Carcharhinus brevipinna* (Gulf of Mexico and Senegal), *Carcharhinus isodon* (Gulf of Mexico), *Carcharhinus limbatus* (Atlantic coast off Florida [hereafter as Florida Atlantic], Gulf of Mexico, and South Carolina), *Carcharhinus* cf. *limbatus* sensu Naylor et al. (2012) (Australia), and *Rhizoprionodon terraenovae* (Florida Atlantic, Gulf of Mexico, South Carolina, and northern Atlantic Ocean) (see fig. 1; supplementary Tables S1 and S2, Supplementary Material online). *Carcharhinus brevipinna*, *C*. *isodon*, and *C*. cf. *limbatus* represent novel host records for *C. gracilis*, and *C*. *limbatus* from Florida Atlantic and from South Carolina and *R*. *terraenovae* from Florida Atlantic and from South Carolina represent novel combinations of host species and locality for *C. gracilis* (see supplementary Table S3, Supplementary Material online). No specimens of *C. gracilis* were recovered from any of the galeocerdid, sphyrnid, triakid, or orectolobiform sharks examined (see supplementary Table S4, Supplementary Material online).

### Single Nucleotide Polymorphism Data Set Generation

In total, eight final filtered single nucleotide polymorphism (SNP) data sets were generated for population genomic analyses (i.e., four data sets for each of the two species). For each species, these include: 1) a data set containing all specimens of sufficient data quality (hereafter a “complete” data set); 2) a complete data set additionally filtered for minimum minor allele count; 3) a complete data set excluding the specimens collected from Senegal (hereafter a “no–Senegal” data set); and 4) a no-Senegal data set additionally filtered for minimum minor allele count. The number of specimens, number of loci, and programs used to analyze each of the eight final filtered SNP data sets are given in supplementary Table S5, Supplementary Material online. For *R. megacantha*, after filtering, 29 (of 39) and 26 (of 34) specimens were retained in the complete and no-Senegal data sets, respectively (supplementary Tables S2 and S5, Supplementary Material online). The specimens retained in the complete data set represented 14 of 19 cownose ray host individuals sampled, and the specimens retained in the no-Senegal data set represented 11 of 14 cownose ray host individuals sampled. Multiple specimens from a single host individual were retained for seven cownose rays for both the complete and no-Senegal data sets (fig. 1*A*, supplementary Table S2, Supplementary Material online). For *C. gracilis*, after filtering, 32 (of 47) and 21 (of 43) specimens were retained in the complete and no-Senegal data sets, respectively (supplementary Tables S2 and S5, Supplementary Material online). The specimens retained in the complete data set represented 15 of 17 requiem shark host individuals sampled, and the specimens retained in the no-Senegal data set represented 14 (of 15) requiem shark host individuals sampled. Multiple specimens from a single host individual were retained for seven requiem sharks for both the complete and no-Senegal data sets (fig. 1*B* and *C*; supplementary Table S2, Supplementary Material online).

### Component Population Structure: *DAPC* and *STRUCTURE*

**Fig. 2.**
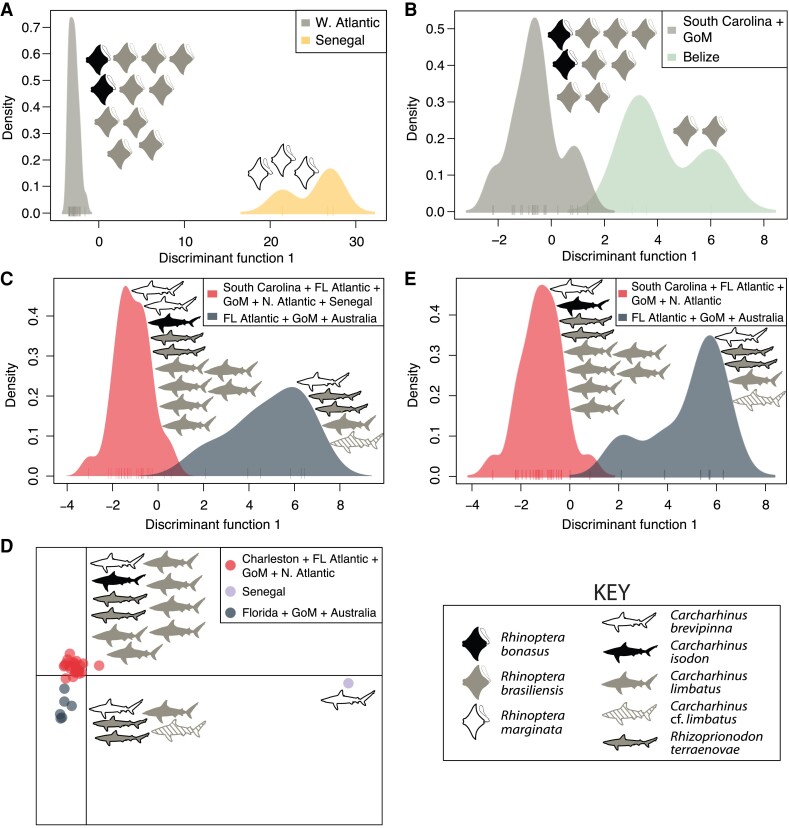
Results of discriminant analysis of principle components (*DAPC*) for *Rhinoptericola megacantha* (*A, B*) and *Callitetrarhynchus gracilis* (*C–E*). Graphic representations of the number of individuals of each host species from which specimens that grouped in each a cluster were collected are presented. Plots (*A*) and (*C, D*) were generated using complete single nucleotide polymorphism data sets not filtered for minor allele count; plots (*B*) and (*E*) were generated using no-Senegal single nucleotide polymorphism data sets not filtered for minor allele count. For both data sets for *R. megacantha* (*A, B*) and for the no-Senegal data set for *C. gracilis* (*E*), AIC and BIC each preferred two clusters. For the complete data set for *C. gracilis*, BIC preferred two clusters (*C*) while AIC preferred three clusters (*D*). FL, Florida; GoM, Gulf of Mexico.

Results from discriminant analysis of principal components (*DAPC*) for both species are presented in figure 2. For analysis of the complete data set for *R. megacantha* with *DAPC*, both Bayesian information criterion (BIC) and Akaike information criteria (AIC) preferred a K-value of 2, separating specimens collected from the eastern Atlantic (i.e., from Senegal; hosted by *R*. *marginata*) from those collected from the western Atlantic (hosted by *R*. *bonasus* and *R*. *brasiliensis*) (fig. 2*A*). A K-value of 2 was also preferred by both AIC and BIC for the no-Senegal data set for *R. megacantha*, separating specimens from Belize (hosted by *R*. *brasiliensis*) from those collected from South Carolina and the Gulf of Mexico (hosted by *R*. *bonasus* and *R*. *brasiliensis*) (fig. 2*B*).

For analysis of the complete data set for *C. gracilis* with *DAPC*, BIC preferred a K-value of 2 (fig. 2*C*) while AIC preferred a K-value of 3 (fig. 2*D*). At K = 2, one cluster comprised six specimens collected from Florida Atlantic, the Gulf of Mexico, and Australia (hosted by *C*. *brevipinna, C*. *limbatus, C*. cf. *limbatus*, and *R*. *terraenovae*), while the second cluster comprised the remaining 26 specimens collected from South Carolina, Florida Atlantic, the Gulf of Mexico, Senegal, and the northern Atlantic Ocean (hosted by *C*. *brevipinna, C*. *isodon, Carcharhinus limbatus*, and *R*. *terraenovae*) (fig. 2*C*). At K = 3, the clustering pattern was identical to that recovered at K = 2, with the exception of the single specimen from Senegal (hosted by *C*. *brevipinna*) recovered in its own cluster (fig. 2*D*). For the no-Senegal data set for *C. gracilis*, a K-value of 2 was preferred, recovering the same two multispecimen clusters described above for the complete data set at K = 3 (fig. 2*E*).

**Fig. 3.**
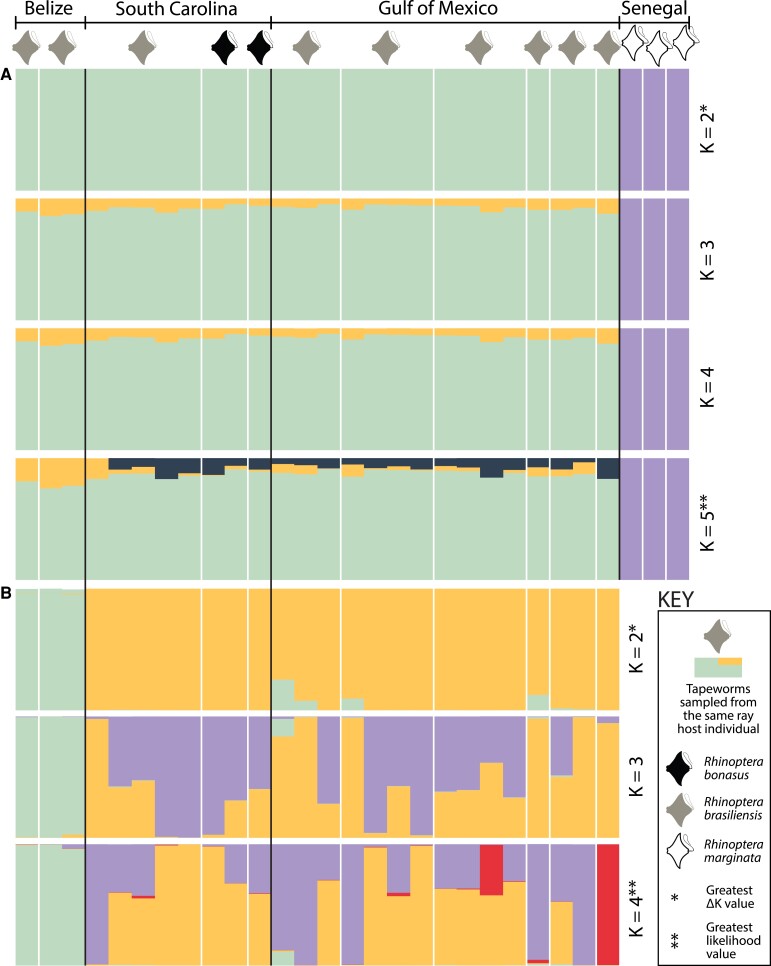
Results of *STRUCTURE* analyses for *Rhinoptericola megacantha* for complete (*A*) and no-Senegal (*B*) single nucleotide polymorphism data sets. Black lines separate sampling localities; white lines separate host individuals within a sampling locality.

Results from *STRUCTURE* analyses for *R. megacantha* are presented in figure 3. For analysis of the complete data set, the Evanno ΔK method preferred a K-value of 2, separating specimens collected from the eastern Atlantic (i.e., from Senegal; hosted by *R*. *marginata*) from those collected from the western Atlantic (hosted by *R*. *bonasus* or *R*. *brasiliensis*) (fig. 3*A*), thus mirroring the results from the *DAPC* analysis. A K-value of 5 had the greatest likelihood value for this data set. Obvious patterns of clustering by host individual, host species, or geographic sampling locality were not revealed by K-values of 3–4 for the specimens collected from the western Atlantic, but at K = 5, the three specimens from Belize are distinguishable from nearly all specimens from the more northern western Atlantic localities. For K-values of 4 and 5, nine of ten *STRUCTURE* replicates binned genetic diversity into only three and four groups, respectively (fig. 3*A*). Results from the single iteration for each of the K = 4 and K = 5 runs that returned genetic diversity binned in as many groups as specified by the K-value are presented in supplementary figure S1, Supplementary Material online. For the single replicate for K = 5 that binned genetic diversity into five groups, the three specimens collected from Belize are distinguishable from the specimens collected from South Carolina and the Gulf of Mexico (supplementary fig. S1, Supplementary Material online). For analysis of the no-Senegal data set for *R. megacantha* with *STRUCTURE*, the Evanno ΔK method preferred a K-value of 2, separating specimens from Belize (hosted by *R*. *brasiliensis*) from those collected from South Carolina and the Gulf of Mexico (hosted by *R*. *bonasus* and *R*. *brasiliensis*) (fig. 3*B*), again mirroring the results of the *DAPC* analysis. No obvious patterns of clustering by host individual, host species, or geographic sampling locality were evident for the specimens collected from South Carolina and the Gulf of Mexico for K-values of 3 and 4 for the no-Senegal data set (fig. 3*B*).

**Fig. 4.**
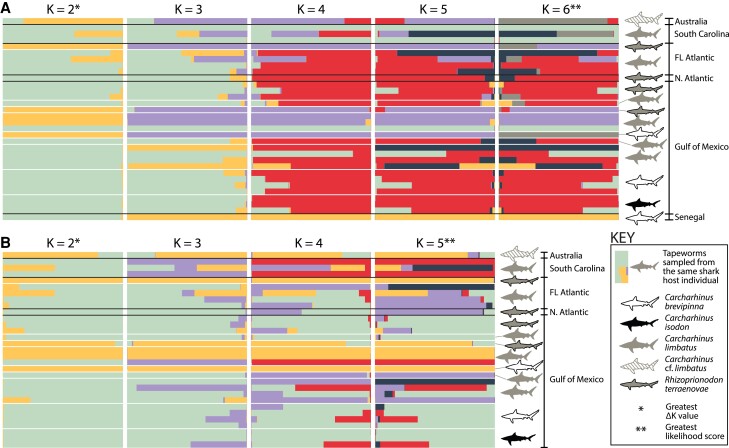
Results of *STRUCTURE* analyses for *Callitetrarhynchus gracilis* for complete (*A*) and no-Senegal (*B*) single nucleotide polymorphism data sets. Black lines separate sampling localities; white lines separate host individuals within a sampling locality. FL, Florida.

Results from *STRUCTURE* analyses for *C*. *gracilis* are presented in figure 4. For analysis of the complete data set, the Evanno ΔK method preferred a K-value of 2, while a K-value of 6 had the greatest likelihood value (fig. 4*A*). For K = 2, the individual from Australia hosted by *C*. cf. *limbatus*, the individual from Florida Atlantic hosted by *R*. *terraenovae*, and four individuals from the Gulf of Mexico hosted by *C*. cf. *limbatus, C*. *brevipinna*, and *R*. *terraenovae* were recovered as sharing similar genetic backgrounds (fig. 4*A*; primarily yellow bars for K = 2), echoing the results of the *DAPC* analyses (fig. 2*C*–*E*; dark blue cluster). These individuals were also consistently recovered as sharing similar genetic backgrounds for K-values 3–5 (fig. 4*A*; primarily purple bars), with the specimen from Australia hosted by *C*. cf. *limbatus* and the specimen from *C*. *brevipinna* from the Gulf of Mexico identified as most similar to one another at K = 6 (fig. 4*A*; primarily gray bars). Additionally, for K-values 4–6, the specimen from Senegal hosted by *C*. *brevipinna* was consistently recovered as distinct from specimens from the western Atlantic and Australia. No obvious clustering by host individual or host species was evident for any K-values tested for the complete data set for *C. gracilis*, but for K-values 4–6, a general pattern of genetic similarity shared between specimens from the Gulf of Mexico, Florida Atlantic, and the northern Atlantic Ocean—to the exclusion of specimens South Carolina, and the group of six specimens discussed above—was evident (fig. 4*A*).

For analysis of the no-Senegal data set for *C. gracilis* with *STRUCTURE*, the Evanno ΔK method preferred a K-value of 2, and the greatest K-value tested (in this case, K = 5) had the greatest likelihood value (fig. 4*B*). Only a single specimen was excluded to create the no-Senegal data set for *C. gracilis*, and overall, comparable patterns of genetic similarity for the remaining specimens at K = 2–5, as described above for the complete data set, were recovered (fig. 4*B*).

### Phylogenetic Analysis: *RAxML*

**Fig. 5.**
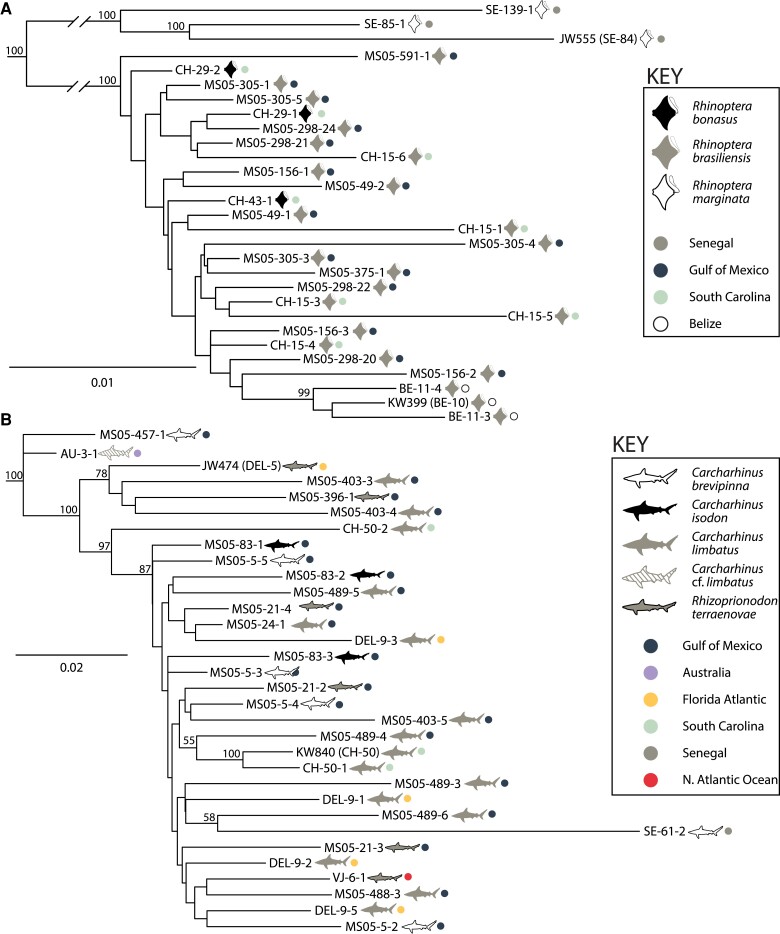
Trees resulting from *RAxML* maximum likelihood phylogenetic analyses for *Rhinoptericola megacantha* (*A*) and *Callitetrarhynchus gracilis* (*B*). Trees are based on complete single nucleotide polymorphism data sets and are rooted to maximize subtree balance. Taxon labels are unique lab specimen numbers comprising unique host accession numbers and specimen numbers, or unique specimen numbers followed in parentheses by host accession numbers and are followed by graphic representations of host species and geographic locality. Nodal support is given as BS values generated from 1,000 rapid BS replicates; BS values <50 are not shown. Scale bars at left indicate nucleotide substitutions per site.

Following concatenation of different loci and removal of invariant sites from complete SNP data sets for both species, final alignment lengths were 1,843 sites for *R. megacantha* and 1,501 sites for *C. gracilis*. The most likely topologies as inferred by *RAxML* are presented in figure 5*A* for *R. megacantha* and figure 5*B* for *C. gracilis*. For *R. megacantha*, the most likely topology showed two well-supported clades with BS ≥99: a clade containing the three specimens from Senegal (hosted by *R*. *marginata*; BS = 100) sister to a clade containing all 26 specimens from western Atlantic (hosted by *R*. *bonasus* and *R*. *brasiliensis*; BS = 100). Within the western Atlantic clade, specimens from Belize (hosted by *R*. *brasiliensis*), formed a well-supported subclade (BS = 99; fig. 5*A*). For *C. gracilis*, beyond the fact that two of the three specimens sequenced from the host individual CH–50 (*C*. *limbatus* from South Carolina) were recovered as sister to one another with strong support (BS = 100), no pattern of grouping by host individual, host species, or geographic sampling locality was evident (fig. 5*B*). The single specimen from Senegal hosted by *C*. *brevipinna* was recovered as subtended on a relatively long branch (fig. 5*B*).

### Population-Level Summary Statistics and Pairwise Distances

**Fig. 6.**
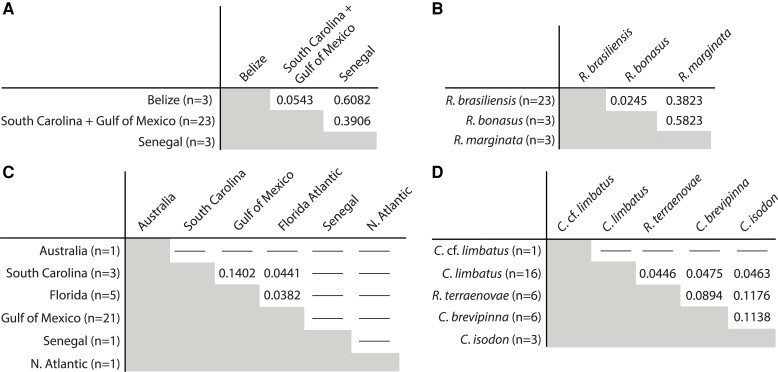
Corrected AMOVA F_ST_ values calculated using the *populations* module of *Stacks* for *Rhinoptericola megacantha* grouped by geographic sampling locality (*A*) and host species (*B*), and for *Callitetrarhynchus gracilis* grouped by geographic sampling locality (*C*) and host species (*D*). The number of tapeworm specimens in each subpopulation is specified in parentheses; F_ST_ values are not reported for comparisons involving subpopulations with only a single individual.

Relevant population-level summary statistics (e.g., number of polymorphic sites, number of private alleles, observed homozygosity, F_IS_, nucleotide diversity [π]) for both species for the four iterations of *populations* run are presented in Table 1, and corrected AMOVA F_ST_ values for both species are presented in figure 6; F_ST_ values for comparisons involving a subpopulation containing a single specimen are not reported. For *R. megacantha*, the three specimens from Senegal (hosted by *R*. *marginata*) had a high proportion of private alleles (i.e., ∼47%) despite comprising a relatively small proportion of the total data set (i.e., three of 29 specimens) (Table 1). Additionally, F_ST_ values were elevated (i.e., ∼0.38–0.61) between specimens of *R. megacantha* from Senegal (hosted by *R*. *marginata*) and other subpopulations in both the by–geography (fig. 6*A*) and by–host species (fig. 6*B*) comparisons. For *C. gracilis*, F_ST_ values ranged from ∼0.04 to 0.14 in the by–geography comparisons (fig. 6*C*) and from ∼0.05 to 0.11 in the by-host species comparisons (fig. 6*D*). Observed homozygosity was elevated (i.e., > 0.91) for all subpopulations tested for both species, and values of F_IS_ ranged from ∼0.01 to 0.2 for *R. megacantha* and from ∼0.03 to 0.23 for *C. gracilis* for all subpopulations containing more than a single specimen (Table 1).

**Table 1:** Population-level Summary Statistics for the Complete Single Nucleotide Polymorphism Data Sets Generated for *Rhinoptericola Megacantha* and *Callitetrarhynchus Gracilis*. For each species, the *populations* module of *Stacks* was run by grouping specimens by either geographic collection locality or host species.

Species	Grouping	Subpopulation	*n*	No. sites analyzed	No. (%) polymorphic sites	No. private alleles (%)	Observed (expected) homozygosity	F_IS_	π
***Rhinoptericola megacantha***									
	Geography	SC and GoM	23	2,568	1,297 (50.51%)	944 (36.76%)	0.94971 (0.9251)	0.17954	0.07689
		Belize	3	2,566	322 (12.55%)	76 (2.96%)	0.94557 (0.95067)	0.01236	0.06116
		Senegal	3	2,537	620 (24.44%)	1,181 (46.55%)	0.91493 (0.89404)	0.07882	0.13043
	Host	Rbon	3	2,568	381 (14.84%)	18 (0.70%)	0.94224 (0.94835)	0.01139	0.06412
		Rbra	23	2,568	1,356 (52.80%)	915 (35.65%)	0.95027 (0.92225)	0.19593	0.07981
		Rmar	3	2,537	620 (24.43%)	1,181 (46.55%)	0.91493 (0.89404)	0.07882	0.13043
***Callitetrarhynchus gracilis***									
	Geography	SC	3	3,866	473 (12.23%)	268 (6.93%)	0.9634 (0.95339)	0.04062	0.06064
		FL Atlantic	5	3,908	866 (22.16%)	513 (13.13%)	0.95347 (0.93322)	0.06801	0.07786
		GoM	21	3,908	2,648 (67.76%)	2,111 (54.02%)	0.96095 (0.92215)	0.23197	0.08064
		N. Atlantic	1	2,972	111 (3.73%)	102 (3.43%)	0.96265 (0.98133)	—	0.03735
		Senegal	1	2,455	131 (5.33%)	236 (9.61%)	0.94664 (0.97332)	—	0.05336
		Australia	1	2,788	146 (5.24%)	90 (3.22%)	0.94763 (0.97382)	—	0.05237
	Host	Cbre	6	3,906	1,009 (25.83%)	639 (16.36%)	0.95662 (0.92667)	0.09787	0.08403
		Clim	16	3,908	2,260 (57.83%)	1,696 (43.4%)	0.96215 (0.91978)	0.22408	0.08388
		Ccflim	1	2,788	146 (5.24%)	90 (3.23%)	0.94763 (0.97382)	—	0.05237
		Ciso	3	3,854	446 (11.57%)	255 (6.62%)	0.95909 (0.9566)	0.0266	0.05703
		Rter	6	3,907	951 (24.34%)	574 (14.69%)	0.95712 (0.93183)	0.07912	0.07827

Cbre, *Carcharhinus brevipinna*; Ccflim, *Carcharhinus* cf. *limbatus*; Ciso, *Carcharhinus isodon*; Clim, *Carcharhinus limbatus*; FL, Florida; GoM, Gulf of Mexico; Rbon, *Rhinoptera bonasus*; Rbra, *Rhinoptera brasiliensis*; Rmar, *Rhinoptera marginata*; Rter, *Rhizoprionodon terraenovae*; SC, South Carolina.

**Fig. 7.**
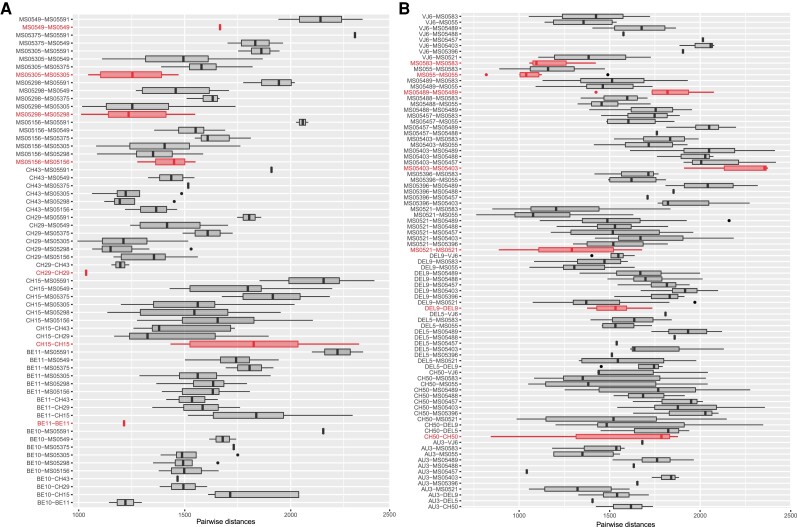
Plots of pairwise distances between tapeworm specimens within an infracommunity and between pairs of infracommunities for *Rhinoptericola megacantha* (*A*) and *Callitetrarhynchus gracilis* (*B*) based on no-Senegal single nucleotide polymorphism data sets. Comparisons within an infracommunity (i.e., between tapeworm specimens from a single host individual) are highlighted.

Results of pairwise distance calculations within and between infrapopulations for both species are presented in figure 7 and supplementary Tables S6 and S7, Supplementary Material online (for the no-Senegal data sets) and supplementary figure S2, Supplementary Material online (for the complete data sets). For both species, only a single specimen from each host collected from Senegal was included in SNP data sets (i.e., one specimen of *R. megacantha* from each of three specimens of *R*. *marginata* from Senegal, and one specimen of *C. gracilis* from one individual of *C*. *brevipinna* from Senegal; see fig. 1). Inclusion of specimens from Senegal—which are genetically divergent from their conspecifics (fig. 5)—in plots of pairwise distances made plots challenging to interpret, and data for only a single tapeworm per host individual did not allow for comparisons of genetic distances within versus between infrapopulations for specimens from Senegal. Thus, results from comparisons of genetic distances between conspecifics within and between infrapopulations are based on plots generated from no-Senegal data sets for both species (fig. 7; supplementary Table S6, Supplementary Material online).

For both *R. megacantha* (fig. 7*A*) and *C. gracilis* (fig. 7*B*), the lowest pairwise distances were recovered between specimens from different infrapopulations (i.e., from different host individuals). Based on range, variability, and statistical comparison, pairwise distances within an infrapopulation were indistinguishable from those representing comparisons between two infrapopulations (supplementary Table S7, Supplementary Material online). For both species, pairwise distances between conspecific specimens within an infrapopulation were as high, or higher, than those between specimens from different infrapopulations (fig. 7; supplementary Tables S6 and S7, Supplementary Material online).

## Discussion

### Definitive Host Specificity and Population-Level Genetic Structure

For this study, species of trypanorhynchs with variable degrees of host specificity were specifically targeted to allow for examination of the impact of specificity on population-level genetic structure. Considering the differing degrees of host specificity for *R. megacantha* and *C. gracilis*, as well as the differing vagilities and distributions of their definitive and intermediate hosts (documented and hypothesized; see discussion, below), it is predictable that less genetic structure was recovered for *C. gracilis* overall (figs. 2–4 and 6, supplementary fig. S2, Supplementary Material online). Relaxed host specificity and increased host vagility should contribute to reduced genetic structure in parasites (Nadler 1995; Huyse et al. 2005). However, few studies to date have comprehensively tested this prediction across comparable species, making it challenging to place these results in context.

Perhaps the most comparable data come from lice. Avian body lice in the genus *Physconelloides* are more host-specific than wing lice in the genus *Columbicola*. Researchers found species of *Physconelloides* to exhibit a greater degree of mitochondrial phylogenetic structure—among both host species and geographic localities—as compared to less host-specific species of *Columbicola* (Johnson et al. 2002; Clayton and Johnson 2003). Similarly, within a species of mouse louse known to house both a host-specific and a nonspecific lineage, a greater degree of isolation by distance was estimated for the host-specific lineage (Martinů et al. 2018). Despite their shared parasitic lifestyles, lice and marine tapeworms have inarguably different biologies and life histories (e.g., arthropods vs. flatworms, terrestrial vs. marine host habitats, single-host vs. multihost life-cycles, direct vs. trophic transmission, etc.) and those differences likely affect patterns of genetic structure. However, the results herein, and those from studies of lice, suggest that host specificity may indeed provide a reliable indicator for expected degree of genetic structure in parasites, and further, that this predictability may hold across a diversity of host-parasite systems.

### *Rhinoptericola megacantha* Component Population-Level Structure: Definitive Host Species Versus Geography

For *R. megacantha*, *DAPC*, *STRUCTURE*, and *RAxML* analyses, population-level summary statistics, and plots of pairwise distances all clearly distinguished specimens collected from the eastern Atlantic (i.e., from *R*. *marginata* in Senegal) from those collected from the western Atlantic (i.e., from *R*. *bonasus* or *R*. *brasiliensis* in South Carolina, the Gulf of Mexico, and/or Belize) (Table 1, figs. 2*A*, 3*A*, 5*A*, and 6*A*; supplementary fig. S2*A*, Supplementary Material online). *Rhinoptera marginata* is restricted to inshore waters from Portugal to Congo (including the Mediterranean Sea) while *R*. *bonasus* and *R*. *brasiliensis* are sympatric for most of their range, collectively the waters are found only off the eastern coasts of North, Central, and South America (Last et al. 2016); none of these three species has been documented from both sides of the Atlantic Ocean. Given that specimens of *R. megacantha* from Senegal and the western Atlantic parasitize allopatric species of cownose ray hosts, it is unsurprising that all analyses based on SNP data suggest that they represent distinct subpopulations. Since the species of cownose ray hosts from which specimens were recovered differ between the two regional localities, however, whether these subpopulation-level differences are driven by definitive host species or geography cannot be reliably disentangled. Nevertheless, specimens sampled from within the western Atlantic do have the potential to address this question.

Within the western Atlantic, results suggest geography to play a more important role than definitive host species in structuring genetic diversity in *R. megacantha*. Rather than recovering specimens from *R*. *brasiliensis* (regardless of their collection locality) more genetically similar to one another than to specimens collected from *R*. *bonasus*, all SNP-based analyses suggest that specimens from *R*. *brasiliensis* from Belize comprise a subpopulation that is genetically distinct from specimens collected from South Carolina and the Gulf of Mexico hosted by either *R*. *brasiliensis* or *R*. *bonasus* (figs. 2*B*, 3*B*, 5*A*, and 6*B*). After filtering, three (of six) specimens of *R. megacantha* hosted by two (of four) individuals of *R*. *bonasus* were included in SNP data sets, versus 20 specimens hosted by seven individuals of *R*. *brasiliensis*. However, only three specimens each were sampled from both Senegal (three host individuals) and Belize (two host individuals), and analyses still recovered distinct genomic signatures for these sets of specimens. Thus, despite limits in sampling, geography—rather than definitive host species—appears to be the primary driver of population structure in *R. megacantha*.

Few studies have probed the population genetics of parasites, and even fewer have focused on tapeworms specifically. There is, however, some evidence from restriction fragment length polymorphism, single locus, and microsatellite data sets to support a predominant role of geography in genetic structure for tapeworms that are not strictly host-specific (Lymbery et al. 1990, 1997; Nakao et al. 2009; Štefka et al. 2009). While findings herein for *R. megacantha* align with those of previous studies, a comprehensive explanation for them may prove challenging. Given that this study is the first of its kind for elasmobranch tapeworms, potential explanations for the observed patterns are worth exploring despite sampling limitations and the lack of knowledge about the life history of *R. megacantha*.

The genetic difference observed between subpopulations on either side of the Atlantic Ocean was expected given the vast distance between these two regions, and, concurrently, the use of a different species of definitive host in each region. Potentially more challenging to explain is the genetic differentiation between specimens from the Caribbean (i.e., Belize) versus more northern collecting localities despite apparent panmixia between subpopulations in the eastern Atlantic and the Gulf of Mexico (figs. 2*A*, 2*B*, and 3*B*). This pattern is intriguing considering studies for a wide range of marine taxa document phylogeographic breaks between populations in the Gulf of Mexico and the Atlantic (Avise 1992; Neigel 2009). Further perplexing is that the three localities in question are all connected by the strong oceanic Loop Current (Geyer et al. 2020) which has the potential to disperse eggs of *R. megacantha* (the only free-living stage of the life cycle) between these localities, and so maintain genetic homogeneity between all three subpopulations.

Additional factors potentially contributing to the patterns of genetic structure observed for *R. megacantha* may thus include definitive host movement, intermediate host distribution and movement, and chemical oceanography, or a combination of these. The observed pattern could be readily explained by cownose rays routinely moving between the Gulf of Mexico and the east coast of the United States, but not between these localities and the Caribbean. However, available data on cownose ray movement suggest that this does not occur—at least for *R*. *bonasus*. Satellite tagging studies have shown that individuals of *R*. *bonasus* migrate south along the Atlantic Coast of the United States in the fall and end their migration to overwinter in waters off the east coast of Florida and do not round the cape of Florida or enter the Gulf of Mexico (Omori and Fisher 2017; Ogburn et al. 2018). Since genetic data similarly suggest that *R*. *bonasus* does not occur in the Gulf of Mexico (Naylor et al. 2012), it seems unlikely that movements of individuals of this host species are responsible for connecting subpopulations of *R. megacantha.* In contrast, *R*. *brasiliensis* has only been recognized to co–occur with *R*. *bonasus* relatively recently (see, e.g., Last et al. 2016), and so little is known about the movements of *R*. *brasiliensis* in the more northern Atlantic region. While individuals of *R*. *brasiliensis* have been reported from off the Atlantic Coast of the United States, including from South Carolina (i.e., the single specimen from South Carolina included in this study; see fig. 1; supplementary Table S1, Supplementary Material online), North Carolina (Naylor et al. 2012), and potentially even as far north as New Jersey (Stoeckle et al. 2020), data on the movement of individuals between the Gulf of Mexico and the Atlantic, or between the Caribbean and the Gulf of Mexico, are lacking.

An alternative hypothesis for population substructure in *R. megacantha* would involve intermediate host distribution and movement. Like all tapeworms, *R. megacantha* has a complex, multi-host life-cycle and is trophically transmitted, meaning its larvae parasitize several intermediate hosts prior to being consumed by the final ray. Unfortunately, the complete natural life cycle of *R. megacantha* has yet to be elucidated, and nothing concrete is known about intermediate host use for this species. Data on partial life-cycles for other species of trypanorhynchs suggest that members of the order almost universally utilize copepods as first intermediate hosts, followed by an invertebrate or vertebrate second intermediate host, and in some cases a vertebrate third intermediate host (Palm 2004). It thus seems likely that *R. megacantha* would utilize a copepod as a first intermediate host, and based on diet data for cownose rays, at least one macroinvertebrate second intermediate host, to complete its life cycle.

The diets of cownose rays in North America have been relatively well-studied (Merriner and Smith 1979; Smith and Merriner 1985). *Rhinoptera bonasus* and *R*. *brasiliensis* appear to be opportunistic generalists with diets dependent on the local abundance of benthic invertebrate prey—specifically bivalves, crustaceans, and polychaetes, as was documented for rays from the Chesapeake Bay (see Smith 1980; Smith and Merriner 1985; Fisher 2010) and the Gulf of Mexico (see Collins et al. 2007; Ajemian and Powers 2012). (Prey composition for cownose rays from the Caribbean is unknown.) If the observed pattern of population structure in *R. megacantha* is attributable to copepod or macroinvertebrate intermediate host movement, rather than cownose ray movement, based on these results, we expect *R*. *megacantha* utilizes different intermediate hosts in the Caribbean versus the northern Atlantic and Gulf of Mexico, and that the intermediate host(s) utilized in the northern Atlantic and the Gulf of Mexico is/are sufficiently vagile to allow for connectivity between the two regions. Spalding et al. (2007) defined marine provinces as areas with distinct biotas and some level of endemism that are bounded by discrete abiotic features. Accordingly, they classified both the northern Gulf of Mexico and the southern and mid-eastern coastal United States as belonging to the same province (i.e., the Warm Temperate Northwest Atlantic), and the Caribbean as a separate province (i.e., the Tropical Northwestern Atlantic). This suggests similar communities of invertebrates between the Gulf of Mexico and Atlantic Coast of the United States, but differences in communities between these regions and Belize. With respect to connectivity between the northern Atlantic and the Gulf of Mexico, survey work on copepods (e.g., Schizas et al. 1999; Drumm and Kreiser 2012) and benthic macroinvertebrates (e.g., Engle and Summers 2000) supports Gulf-to-Atlantic distributions for some taxa, though phylogeographic studies for a wide variety of species have documented distinct genetic differences between populations in the two regions, suggesting little connectivity through movement of individuals even for vagile species (e.g., Drumm and Kreiser 2012). This is likely due to the extent of the Florida peninsula into subtropical waters, creating a biogeographic barrier to dispersal (Avise 1992). More information on intermediate host use in *R. megacantha* is clearly needed.

### *Rhinoptericola megacantha* Infrapopulation Structure: Genetic Diversity Within Versus Between Host Individuals

For the seven instances where SNP data for more than one specimen of *R. megacantha* from a single host individual could be analyzed, comparisons of pairwise distances clearly demonstrate that similar levels of genetic diversity can be found within infrapopulations as between infrapopulations (fig. 7*A*; supplementary Tables S6 and S7, Supplementary Material online). Similarly, *STRUCTURE* and *RAxML* results suggest that infrapopulations comprise specimens that encompass a mix of genetic backgrounds (figs. 3*B* and 5*A*). Unfortunately, comparable data for other species of trypanorhynchs are not available. To date, the work that has been done to examine intraspecific genetic divergence in trypanorhynchs has been restricted to data for only single loci (e.g., partial 28S rDNA, COX1) and has focused mainly on establishing boundaries between intra- and interspecific divergence. The results of this body of work are summarized by Herzog and Jensen (2022). Comparable data from other species of tapeworms, however, suggest that infrapopulations comprised of genetically dissimilar individuals (i.e., “mixed infections”) are not uncommon (e.g., Lymbery and Thompson 1989; Nakao et al. 2003; Pajuelo et al. 2017). Theory and evidence to support why genetically heterogenous infrapopulations in definitive hosts should be expected for parasites with complex life-cycles have been advanced. For example, Rauch et al. (2005) used microsatellite markers to characterize the population genetics of the eye-fluke *Diplostomum pseudospathaceum* (Class Trematoda: Order Diplostomida) and found that, in natural populations, the majority of *D. pseudospathaceum* released from a single snail first intermediate host were genetic clones, but that fish second intermediate hosts were typically infected with a diverse array of eye-fluke genotypes. They therefore argued for the maintenance of more than one intermediate host in a parasite's life-cycle as a strategy for increasing genetic diversity at the level of the definitive host, thereby reducing the risk of inbreeding during a reproductive event. Thus, the presence of genetically diverse infrapopulations of *R. megacantha* in single cownose rays likely results from gradual accumulation of dissimilar tapeworm genotypes within host individuals at each subsequent step in the life cycle.

### *Rhinoptericola megacantha* Population-Level Summary Statistics

The large proportion of specimens (i.e., 23 of 29) collected from *R*. *brasiliensis—*and specifically the 20 specimens from this species from the Gulf of Mexico—encompassed the greatest proportion of genetic diversity sampled for *R. megacantha*, as reflected in high proportions of polymorphic sites and private alleles reported for the subpopulations that include these specimens (Table 1). Filtering led to the inclusion of only three specimens each in the Belize and Senegal subpopulations (in the by–geography comparison), and in the *R*. *bonasus*–hosted and *R*. *marginata*–hosted subpopulations (in the by–host species comparison). Thus, population summary statistics—and particularly F_ST_ values—for each of these subpopulations should be evaluated with caution. Nevertheless, the F_ST_ values reported herein are corrected AMOVA F_ST_ values, and are thus not biased by differences in sample sizes between subpopulations, and recent simulations show that F_ST_ values are not artificially inflated by small sample sizes if the number of biallelic markers sampled is sufficiently great (i.e., multiple thousands of loci, as sampled herein) (Willing et al. 2012). In all, summary statistics and F_ST_ values for possible subpopulations of *R. megacantha* seem to support the same patterns as *DAPC*, *STRUCTURE*, and *RAxML* analyses: Distinct subpopulations on either side of the Atlantic Ocean and more apparent differentiation between Belize and the Gulf of Mexico + South Carolina than exists between specimens collected from *R*. *bonasus* versus *R*. *brasiliensis* (Table 1, fig. 6*A* and *B*).

Though investigating the mating systems of trypanorhynch tapeworms was beyond the scope of this study, it is interesting to note that consistently high levels of homozygosity (i.e., > 0.91) and positive F_IS_ values were recovered across subpopulations of *R. megacantha* (Table 1). The few studies available for comparison corroborate these findings as typical for tapeworms, and suggest they indicate some degree of selfing and/or inbreeding (Lymbery et al. 1990, 1997; Binz et al. 2000; Nakao et al. 2003). In fact, comprehensive assessments of inbreeding and mating system structure for the tapeworm *Oochoristica javaensis* found that selfing, kin-mating, and outcrossing all contribute to reproduction (Detwiler et al. 2017; Detwiler and Criscione 2017).

### *Callitetrarhynchus gracilis* Component Population-level Structure: Definitive Host Species Versus Geography

For *C. gracilis*, results from *DAPC*, *STRUCTURE*, and *RAxML* analyses indicated no apparent genomic structure aligned with definitive host species or geographic locality beyond a distinction in some analyses between the single specimen collected from Senegal and its remaining 31 conspecifics collected from Australia or the western Atlantic (figs. 2*D* and 4*A*; supplementary fig. S2*B*, Supplementary Material online). However, comparisons of pairwise distances within versus between infrapopulations of *C. gracilis* that include the specimen from Senegal clearly showed that, while this specimen is genetically dissimilar from its conspecifics, the degree of dissimilarly is not nearly as extreme as observed for the three specimens of *R. megacantha* collected from Senegal (see supplementary fig. S2*B*vs. 2*A*, Supplementary Material online).

Regrettably, no specimens of *C. gracilis* from Belize were available to assess whether the same pattern of genomic differentiation between the Caribbean and the Gulf of Mexico and western Atlantic observed for *R. megacantha* also holds for *C. gracilis*. It is worth noting, however, that the single specimen of *C. gracilis* collected from Australia was indistinguishable from those collected from the western Atlantic based on SNP data.

The circumglobal distributions and apparently high vagilities of both the definitive and intermediate hosts of *C. gracilis* likely contributed to the low level of differentiation found across the broad range of host species and geographic regions sampled. Even considering only the five species of requiem sharks parasitized by the specimens of *C. gracilis* sequenced here (an overly conservative estimate of its actual range of suitable definitive hosts; see supplementary Table S3, Supplementary Material online), *C. gracilis* parasitizes shark species that each have broad geographic ranges (e.g., from occurring circumglobally in warm temperate to tropical waters like *C*. *brevipinna* to a range spanning the Caribbean, Gulf of Mexico, and eastern coast of the United States such as *R*. *terraenovae*) (Gallo et al. 2010; Naylor et al. 2012; Ebert et al. 2021). Parasitizing the five species of requiem sharks sampled in this study alone qualifies *C. gracilis* as potentially circumglobally distributed in temperate and tropical oceans.

A broad geographic range for a species is, however, not necessarily indicative of the movement of individuals. Data on the movements of individual sharks are available for four of the five species of sharks represented in this study. Movement data for individuals of *C*. *brevipinna* (*n* = 1,750), *C*. *isodon* (*n* = 2,865), *C*. *limbatus* (*n* = 10,551), and *R*. *terraenovae* (*n* = 5,055) suggest that these four species are quite vagile across their range (or at least within the waters off the Eastern Seaboard) (Kohler and Turner 2018). The limited genetic divergence and structure recovered herein for *C. gracilis* may thus be attributable to movements of its definitive shark hosts.

As for *R. megacantha*, it is important to consider the complex, multihost life cycle of *C. gracilis*, for the distributions and movements of intermediate hosts may be just as important, if not more important, than those of definitive hosts when considering tapeworm population structure. Palm (1997, 2004) hypothesized a four-host life-cycle for *C. gracilis*, with copepods servings as first intermediate hosts, clupeids (herrings) serving as second intermediate hosts, scombrids (mackerels and tunas) and epinephelids (groupers) serving as third intermediate hosts, and carcharhinids (requiem sharks) serving as definitive hosts. Unlike *R. megacantha*, for which no data on intermediate host use are available, reports of larvae of *C. gracilis* abound in the literature. Over 140 species of teleosts representing tens of families worldwide have been reported as intermediate hosts of *C. gracilis* (see Palm 2004). Even if some of these reports constitute misidentifications (see Palm 2004), the intermediate host use of *C. gracilis* would still be impressively broad. For example, clupeids and epinephelids are found worldwide in tropical to temperate coastal waters, and scombrids are globally distributed in the marine tropics and subtropics (Hastings et al. 2015; Nelson et al. 2016), and scombrids are known for their expansive ranges and migratory behavior. In particular, the Atlantic bluefin tuna (*Thunnus thynnus*)—which has been reported as an intermediate host of *C. gracilis* (see Palm 2004)—is known to migrate great distances and exhibits little genetic structure between the Atlantic and Mediterranean (Nelson et al. 2016). The pattern of limited genetic structure recovered for *C. gracilis* herein aligns with a life-cycle involving large, vagile, wide-ranging host species like scombrids and carcharhinids.

In general, SNP-based analyses support a model of little population-level structure in *C. gracilis*. However, the *DAPC* analysis using AIC over BIC to inform clustering patterns (fig. 2*D*), the higher K-values tested in *STRUCTURE* analyses (fig. 4*A*), and the comparisons of pairwise distances between infrapopulations (supplementary fig. S2*B*, Supplementary Material online) all revealed genetic differentiation between the single specimen collected from Senegal and its conspecifics while indicating little differentiation between specimens collected from the western Atlantic and Australia. Unlike for *R. megacantha*, which utilizes a different species of definitive host in Senegal as compared to the western Atlantic, the single specimen of *C. gracilis* from Senegal included in SNP-based analyses came from a host species represented by individuals collected from both the western and eastern Atlantic (fig. 1, supplementary Table S1, Supplementary Material online). Given this replication within a host species and across geographic regions, any observed differences—if not attributable to small sample size—seem more likely to be driven by oceanographic or biogeographic barriers as opposed to differences in definitive host species. Ultimately, an explanation for why a specimen from the eastern Atlantic Ocean is genetically distinct from specimens collected from the western Atlantic and Indian Oceans when specimens from the latter subpopulations appear largely undifferentiated is challenging. A more complete picture of intermediate and definitive host use by *C*. *gracilis*, and more comprehensive sampling of specimens across the circumglobal range of this species, are needed to better understand this result.

For species of tapeworms with wide geographic ranges and relaxed host specificity, patterns of limited genetic structure have been similarly documented (Brabec et al. 2016; Addy et al. 2017; Fraija-Fernández et al. 2021). Thus, the results for *C. gracilis* seem to follow previously documented patterns.

### *Callitetrarhynchus gracilis* Infrapopulation Structure: Genetic Diversity Within Versus Between Host Individuals

For the seven instances where SNP data for more than a one specimen of *C. gracilis* from a single host individual could be analyzed, comparisons of pairwise distances within and between infrapopulations and results from *DAPC* and *STRUCTURE* suggest that specimens within an infrapopulation are not necessarily more genetically similar to one another than are specimens between infrapopulations (figs. 2*C*–*E*, 4, and 7*B*, supplementary fig. S2*B*, Supplementary Material online). Additionally, specimens collected from the same host individual were almost never recovered as one another's closest relatives in the *RAxML* analysis (fig. 5*B*), further supporting this assessment. These results for infrapopulation-level genetic diversity for *C. gracilis* essentially mirror those recovered for *R. megacantha*. Possible explanations, including context from other tapeworm systems, are discussed in detail for *R. megacantha*; those considerations are similarly applicable for *C. gracilis* (see above).

A slightly wider range of pairwise distances within and between infrapopulations was recovered for the no-Senegal data set for *C. gracilis* as compared to *R. megacantha* (fig. 7, supplementary fig. S2, Supplementary Material online). This is potentially due to the slightly greater number of specimens included in no-Senegal SNP data sets for *C. gracilis* as compared to *R. megacantha* (i.e., 29 vs. 26, respectively; see supplementary Table S5, Supplementary Material online) and the greater number of host individuals from which infrapopulations could be compared (i.e., 13 sharks rays vs. 11 rays in no-Senegal data sets; see fig. 1, supplementary Table S2, Supplementary Material online). It may alternatively indicate that this subpopulation of *C. gracilis* truly boasts more standing genetic variation than does that of *R. megacantha*.

### *Callitetrarhynchus gracilis* Population-Level Summary Statistics

The large proportion of specimens collected from the Gulf of Mexico (i.e., 21 of 32) and those from *C*. *limbatus* (i.e., 16 of 32) encompassed the greatest proportion of genetic diversity sampled for *C. gracilis* in the by–geography and by–host species comparisons, respectively. High proportions of polymorphic sites and private alleles were recovered for both of these purported subpopulations (Table 1). Sampling limitations or data filtering (or a combination thereof) led to low samples sizes for South Carolina, the northern Atlantic Ocean, Senegal, and Australia subpopulations in the by–geography comparisons, and for the *C*. cf. *limbatus–*hosted and *C*. *isodon*–hosted subpopulations in the by–host species comparisons. As discussed for *R. megacantha*, population summary statistics—and particularly F_ST_ values—for these subpopulations should thus be evaluated cautiously (see above). Regardless, summary statistics and F_ST_ values for possible subpopulations of *C. gracilis* generally support the same pattern as *DAPC*, *STRUCTURE*, and *RAxML* analyses (i.e., limited subpopulation-level structure by definitive host species or geographic region; fig. 6*C* and *D*, Table 1).

Additionally, consistently high levels of homozygosity (i.e., > 0.94) and positive F_IS_ values were found for all subpopulations of *C. gracilis* (Table 1), mirroring the results for *R. megacantha*. Context from other tapeworm systems on expected levels of homozygosity and potential levels of selfing and/or inbreeding is provided and discussed for *R. megacantha*, and those same considerations are relevant for *C. gracilis* (see above).

### High Rates of Homozygosity and *STRUCTURE*

The high levels of homozygosity (i.e., > 91%) inferred for both *R. megacantha* and *C. gracilis* (see Table 1) have the potential to bias the results of *STRUCTURE* analyses. A hallmark of unaccounted for subpopulation structure is fewer heterozygotes in a population than would be expected under Hardy–Weinberg equilibrium (HWE) (Wahlund 1928). *STRUCTURE* operates by binning genetic data into *n* = K clusters such that deviations from HWE within each bin are minimized. *STRUCTURE* also assumes random mating and therefore assumes that lower–than–expected heterozygosity is the result of unaccounted for substructure. Thus, for hermaphroditic species like tapeworms, in which reduced heterozygosity could be expected as a result of high rates of selfing, there is the risk for *STRUCTURE* to infer subpopulation structure that is not biologically real (Falush et al. 2003; Gao et al. 2007). However, for both species, the results from *STRUCTURE* align with the results inferred from *DAPC* and *RAxML* analyses, population-level summary statistics, and plots of pairwise distances in all cases. Additionally, the Evanno ΔK method preferred a K-value of 2 for all *STRUCTURE* analyses (figs. 3 and 4) (i.e., preferred the lowest degree of substructure possible among individuals). Thus, though results from *STRUCTURE* may have been biased by high levels of homozygosity, the overall conclusions drawn about population structure for both species are based on concordant results from a variety of analyses, all of which support the same results inferred by *STRUCTURE*.

## Conclusions

This study is the first to leverage genome-scale data to investigate the correlation between host specificity and geographic range and parasite population generic structure. Component population-level genetic structure in *R. megacantha* was found to correspond to geographic region rather than definitive host species, and component populations of *C. gracilis* were found to have relatively little genetic structure, suggesting a positive correlation between degree of host specificity and degree of genetic structure in trypanorhynchs. Conspecific trypanorhynchs collected from the same host individual were not one another's closest relatives, highlighting the potential importance of second and/or third intermediate hosts in maintaining genetic diversity at the infrapopulation level for tapeworms. High levels of homozygosity (>91%) and positive F_IS_ values for both species suggest in both cases that individuals commonly engage in inbreeding, including even kin–mating and/or selfing. Finally, despite its reputation as an extremely euryxenous species, adults of *C. gracilis* may be restricted to species of requiem sharks (family Carcharhinidae) as definitive hosts.

As discussed, limited sampling necessitates cautious interpretations of these first insights into the population genomics of trypanorhynch tapeworms. Nevertheless, the results of this study mirror those from studies of other species of tapeworms and other parasite-host systems. The role of intermediate hosts in creating and/or maintaining genetic structure in trypanorhynch populations should not be underestimated, and moving forward, studies that reveal whether patterns of structure observed at the level of the definitive host are maintained in line with, or in spite, of intermediate host use will be compelling. Ultimately, the results of this study provide a foundation on which future investigations of marine tapeworm populations genomics can build. This work also supplements the growing body of literature highlighting the complex interplay between biological, ecological, and oceanographic factors in structuring marine parasite populations.

## Materials and Methods

### Sampling Strategy and Specimen Collection

To assess genetic structure of infrapopulations (sensu Bush et al. 1997), multiple conspecific trypanorhynchs were sampled from individual sharks and rays, for up to five tapeworms per host specimen. To assess the structure of trypanorhynch populations across their known host species and geographic ranges (i.e., the structure of a component population), conspecific trypanorhynchs from multiple host individuals of the same host species, both within and across geographic regions, were sequenced. This process was replicated for as many host species and geographic localities as was possible (see fig. 1).

Elasmobranch intestines or intestinal contents fixed in 95% ethanol resulting from global collections of tapeworms made over recent decades were examined for tapeworms. Prior to examination, spiral intestines had been removed from the body cavity and opened with a longitudinal incision. Intestines and their contents were either fixed entirely in 95% ethanol, or intestines were fixed in 10% seawater-buffered formalin and a portion of their contents (including tapeworms) were fixed in 95% ethanol. All ethanol-fixed material was transferred to the University of Kansas (KU) or the University of Connecticut (UConn) and stored in a freezer prior to examination for tapeworms.

For *R. megacantha*, sampling was informed by the comprehensive summary of its known host species and geographic localities compiled by Herzog and Jensen (2022) (see supplementary Table S3, Supplementary Material online). For *C. gracilis*, sampling was informed by reports from the literature of adults from elasmobranchs, summarized herein in supplementary Table S3, Supplementary Material online. In total, material from 19 cownose rays and 165 carcharhiniform sharks was examined (see supplementary Table S4, Supplementary Material online).

For each host individual from which tapeworms included in this study were recovered, the unique host specimen number (e.g., BE-10), disk width (rays) or total length (sharks), sex, collection date, collection locality, number of tapeworms recovered, and number of tapeworms recovered that were ultimately included in final data sets are given in supplementary Table S1, Supplementary Material online. Unique host specimen numbers can be used to search the Global Cestode Database (www.elasmobranchs.tapewormdb.uconn.edu) (Caira et al. 2022) to access additional information about each host. For most hosts individuals, identifications were confirmed by Naylor et al. (2012) using sequence data for the NADH2 gene; for others, NADH2 sequence data were generated from liver tissue preserved in 95% ethanol and sequenced at UConn following the methods outlined in Fernando et al. (2019) (Caira et al. unpublished data; see supplementary Table S1, Supplementary Material online).

Maps of sampling localities with the number of individuals of each host species sampled from each locality, the number of tapeworms recovered from each host individual, and the number of tapeworms recovered that were ultimately included in finalized data sets are provided in figure 1. To visualize sampling localities, geographic coordinates were plotted to maps in *R* v. 4.0.3 (R Core Team [2020]. R: A language and environment for statistical computing. R Foundation for Statistical Computing, Vienna, Austria. https://www.Rproject.org/) via *RStudio* v. 1.3.1093 (RStudio Team [2020]. RStudio: Integrated Development for R. RStudio. PBC, Boston, MA, USA. http://www.rstudio.com/) using the packages *maps* v. 3.4.0 (Becker et al. 2021), *mapdata* v. 2.3.0 (Becker et al. 2018), *maptools* v. 1.12 (Bivand and Lewin-Koh 2022), and *scales* v. 1.1.1 (Wickham and Seidel 2020). Ecological terminology used to define levels of tapeworm populations follows Bush et al. (1997). Ray taxonomy follows Last et al. (2016). Shark taxonomy follows Naylor et al. (2012) and Ebert et al. (2021).

Collections in Australia were made under the auspices of Richard Mounsey and Julie Lloyd, formerly of Darwin Fisheries. Material from Belize was collected under permit no. 00001612 issued to Janine N. Caira, Kirsten Jensen, Fernando P.L. Marques, and Roy Polonio by Fisheries Administrator Beverly Wade of the Belize Fisheries Department (Ministry of Forestry, Fisheries and Sustainable Development), Belize. Material from Florida was collected under the auspices of the University of Southern Mississippi Gulf Coast Research Laboratory and/or the National Marine Fisheries Service, Southeast Fisheries Science Center, Panama City, FL, USA. Collections in the Gulf of Mexico were made under the auspices of the University of Southern Mississippi Gulf Coast Research Laboratory. Material from the northern Atlantic Ocean was collected under the auspices of the National Oceanic and Atmospheric Administration and the National Marine Fisheries Service. Material from Senegal was collected under permit no. 006087 issued by the Ministère de L’Éducation, Dakar, Senegal. Collections in South Carolina were made under the auspices of the South Carolina Department of Natural Resources (Bryan Frazier and Ashley Shaw) and the College of Charleston (Isaure de Buron).

### Specimen Vouchering and DNA Extraction

Prior to DNA extraction, each tapeworm was photographed using a Lumenera INFINITY3–6UR 6.0 megapixel USB 3 microscopy camera (Teledyne Lumenera, Ottawa, ON, Canada) attached to a Leica MZ16 dissecting microscope (Leica Microsystems, Buffalo Grove, IL, USA). Microscissors were used to remove a piece of the strobila and/or scolex of each specimen for DNA extraction. To ensure accurate genotyping, DNA was intentionally not extracted from gravid proglottids (i.e., proglottids containing eggs). Methods for DNA extraction follow Herzog and Jensen (2022). Whole-mounted hologenophores sensu (Pleijel et al. 2008) were then generated from the remaining portions each specimen not utilized for DNA extraction. Methods for hologenophore preparation follow Herzog and Jensen (2018). Hologenophores are deposited at the Lawrence R. Penner Parasitology Collection (LRP) Department of Ecology and Evolutionary Biology, University of Connecticut, Storrs, CT, USA; museum deposition numbers for each hologenophore are provided in supplementary Table S2, Supplementary Material online.

### Restriction Enzyme Selection, Library Preparation, and Next Generation Sequencing

Restriction enzyme selection for the generation of multiplexed shotgun genotyping (MSG) data sets was informed by virtual simulation of enzyme digestion using custom python scripts (courtesy of J.K. Kelly, KU) in *Python* v. 2.7.16 (Van Rossum and Drake 2009). The cut site sequences for the restriction enzymes AseI, Bfal, CviQI, MseI, and NdeI (New England BioLabs, Ipswich, MA, USA) were tested using draft genomes for *Rhinoptericola megacantha* (∼351 Mb) and *Pseudolacistorhynchus heroniensis* (∼1 Gb) (Caira, Jockusch, Ralicki, Wegryzn, and Jensen, unpublished data). As a genome for *C. gracilis* is not available, the draft genome of *P*. *heroniensis—*a member of the same family, Lacistorhynchidae—was used. Results of virtual digestions indicated MseI as the most suitable enzyme based on cut site frequency in repetitive versus nonrepetitive genomic regions and the size distribution of fragments generated. Physical test digestions using MseI were then performed at the University of Kansas Genome Sequencing Core (KU GSC) using extracted genomic DNA for one specimen of each species of interest (i.e., CH-15-6 for *R. megacantha* and MS05-21-4 for *C. gracilis*; see supplementary Table S2, Supplementary Material online). Digested DNA was cleaned up and concentrated using a bead clean up protocol with 2 × AMPure XP beads (Beckman Coulter Life Sciences, Indianapolis, IN, USA), quality checked using an Invitrogen Qubit assay (ThermoFisher Scientific, Waltham, MA, USA), and run on a TapeStation 2200 gDNA screentape (Agilent, Santa Clara, CA, USA) to visualize the proportion of DNA within a given size range.

Extracted genomic DNA for all specimens was used to generate two MSG libraries using MseI and the protocol of Andolfatto et al. (2011) with the following modifications: 1) Unique in-line barcodes were ligated to digested DNA prior to pooling to allow for bioinformatic identification of each specimen after sequencing; and 2) following bead purification, pooled libraries were run on a Blue Pippin 2% agarose gel cassette (Sage Science, Beverly, MA, USA) to elute DNA fragments within a 300–400 bp range. Each of the two multiplexed libraries was sequenced on a single flow cell of an Illumina NextSeq 550 High Output Next Generation Sequencer (Illumina, San Diego, CA, USA) for 75 bp single end reads. All specimens were included in the first library. The second library consisted of a subset of specimens for which insufficient read counts were generated following the first round of library preparation and sequencing (see supplementary Table S2, Supplementary Material online). Library preparation and sequencing was completed at the KU GSC.

### SNP Processing and Filtering

To separately generate single nucleotide polymorphism (SNP) data sets from raw next generation sequence data for each of the two species of interest, *Stacks* v. 2.53 (Catchen et al. 2013; Rochette et al. 2019) was used. Specimens were demultiplexed and low-quality reads and adaptor contamination were removed using the *process_radtags* module with the –*r*, –*c*, and –*q* flags specified. Further filtering was performed using *Trimmomatic* v. 0.39 (Bolger et al. 2014) to remove remaining low-quality reads and adaptor contamination, and to enforce a consistent read length of 70 bp. Read quality was visualized using *FastQC* v. 0.11.7 (Andrews 2010) and *MultiQC* v. 1.7 (Ewels et al. 2016). Throughout the process of SNP data set generation, file conversion was accomplished using *PGDSpider* v. 2.1.1.5 (Lischer and Excoffier 2012) unless otherwise stated.

### SNP Data Set Generation: *Rhinoptericola megacantha*

Availability of the above-mentioned reference genome for *R*. *megacantha* allowed for use of a reference-guided alignment approach in *Stacks*. An index database was built from the reference genome using *Bowtie 2* v. 2.3.5.1 (Langmead and Salzberg 2012). For each of the 39 specimens, demultiplexed and quality filtered reads were aligned to the index using *Bowtie 2* with the ––*no-unal* and ––*sensitive* flags. The program *SAMtools* v. 1.9 (Li et al. 2009) was used to convert the generated sequence alignment map (SAM) files to binary alignment map (BAM) files and to subsequently sort BAM files. The *gstacks* module of *Stacks* was used to generate a by-locus data set, and to genotype specimens at each SNP for each locus using default settings. The *populations* module of *Stacks* was used to export two unfiltered SNP data sets as variant call format (VCF) files using the ––*vcf* flag: one data set containing all 39 specimens (hereafter the “complete” data set) and one data set excluding the six specimens collected from Senegal (hereafter the “no–Senegal” data set) (see supplementary Table S2, Supplementary Material online). For both data sets, all specimens were specified as belonging to a single population in the *populations* module.

Filtering of the complete and no-Senegal SNP data sets was performed iteratively based on various metrics of data quality and completeness in *R* v. 4.0.3 via *RStudio* v. 1.3.1093 using the packages *SNPfiltR* v 0.1.1 (DeRaad 2022) and *VCFR* v. 1.12.0 (Knaus and Grünwald 2017). For both data sets, minimum and maximum read depths of 6 and 100, respectively, and a minimum genotype quality score of 30, were enforced. All loci were constrained to be biallelic, and heterozygous genotypes falling outside of an allele balance range of 0.25–0.75 were excluded. Ten specimens with ≥90% missing data (of 39 specimens) were removed from the complete data set, and seven specimens with ≥90% missing data (of 33 specimens) were removed from the no-Senegal data set (see supplementary Table S2, Supplementary Material online). For both data sets, a SNP completeness cutoff of 80% was specified to ensure no retained specimen had >50% missing data. Linkage disequilibrium between loci was minimized by enforcing a minimum distance of 10,000 bp between SNPs following estimates of linkage disequilibrium decay by Branca et al. (2011). Filtered versions of the complete data set and the no-Senegal data set were then exported. An additional filtering step to remove singletons by enforcing a minimum minor allele count of 3 was then employed following Linck and Battey (2019) for both data sets. These minimum minor allele count–filtered versions of the complete and no-Senegal data sets were then exported.

### SNP Data Set Generation: *Callitetrarhynchus gracilis*

As a reference genome is not available for *C*. *gracilis*, the *Stacks* de novo approach was used for this species. After demultiplexing and initial quality filtering, parameter testing was performed in *Stacks* using the *denovomap.pl* wrapper. Fifteen of the 47 specimens of *C. gracilis* sequenced were chosen for parameter testing based on possession of high read counts and representation across the range of geographic localities and host species sampled. Following Paris et al. (2017), values for the number of raw reads required to form a stack in the *ustacks* module (–*m*), the number of mismatches allowed between stacks to merge them into a putative locus in the *ustacks* module (–*M*) and the number of mismatches allowed between putative loci during catalog construction in the *cstacks* module (–*n*) were varied. In addition to default settings (i.e., –*m 3*, –*M 2*, –*n 1*), values of –*m* (*2*, *4*, *6*, *8* 1*0*) (–*M 2*, –*n 1*) and –*n* –*M* (*2*, *4*, *6*, *8*) (–*m 3*) were tested, with the *minsamplesperpop 0.80* flag employed and all specimens specified as belonging to a single population. Following parameter testing, –*m 4*, –*M 4*, –*n 4* was determined to be the most optimal combination of parameter settings.

Following Cerca et al. (2021), specimens with high proportions of missing data (i.e., “bad apples”) were then identified. Briefly, the *populations* module of *Stacks* was used generate exploratory SNP data sets, both with the –*r 0.4* flag enforced and without an –*r* flag specified, for each of the following combinations of specimens: 1) all 47 specimens, 2) the six specimens collected from South Carolina, 3) the seven specimens collected from Florida Atlantic and the northern Atlantic Ocean, 4) the 29 specimens collected from the Gulf of Mexico, and 5) the four specimens collected from Senegal (see supplementary Table S2, Supplementary Material online). For each iteration of *populations*, each combination of specimens was specified as comprising a single population. Proportions of missing data and mean depth of coverage for each specimen in each of the ten resulting exploratory data sets were assessed separately using the ––*missingindv* and ––*depth flags* in *VCFtools* v. 0.1.16 (Danecek et al. 2011). Eleven specimens were designated as “bad apples” based on high proportions of missing data and low mean depths of coverage across analyses, and were excluded from future data sets (see supplementary Table S2, Supplementary Material online).

Demultiplexed and initially quality filtered reads for the 36 specimens of *C. gracilis* retained (i.e., the “good apples”) were assembled into stacks using the *ustacks* module with the –*m 4*, –*M 4*, and ––*deleverage* flags specified. The *cstacks* module was then used to create a catalog of consensus loci with the *n 4* flag and default settings. The *sstacks*, *tsv2bam*, and *gstacks* modules were used with default settings to match individual stacks to the catalog, transpose data from orientation by specimen to orientation by locus, and genotype specimens at each SNP for each locus. The *populations* module was used to export two unfiltered SNP data sets as VCF files using the ––*vcf* flag: one “complete” data set containing all 36 specimens, and one “no-Senegal” data set excluding the three specimens collected from Senegal (see supplementary Table S2, Supplementary Material online). For both data sets, all specimens were specified as belonging to a single population in the *populations* module.

As for *R. megacantha* (see above), additional filtering was performed iteratively in *R* v. 4.0.3 via *RStudio* v. 1.3.1093 using the packages *SNPfiltR* and *VCFR* v. 1.12.0. For both data sets, minimum and maximum read depths of 5 and 100, respectively, and a minimum genotype quality score of 30, were enforced. All loci were constrained to be biallelic, and heterozygous genotypes falling outside of an allele balance range of 0.25–0.75 were excluded. Four additional specimens with ≥96% missing data (of 36 retained specimens) were removed from the complete data set and two additional specimens with ≥96% missing data (of 33 retained specimens) were removed from the no-Senegal data set (see supplementary Table S2, Supplementary Material online). For both data sets, a SNP completeness cutoff of 65% was specified to ensure no retained specimen had >50% missing data, and only a single SNP per locus was retained. Filtered versions of the complete data set and the no-Senegal data set were then exported. As with *R. megacantha*, additional versions filtered by enforcing a minimum minor allele count of 3 were then generated and exported for both the complete data set and the no-Senegal data set.

### Population Genomic Methods

Population genetic structure was assessed using discriminant analysis of principal components (*DAPC*) (Jombart et al. 2010) implemented in *R* v. 4.0.3 via *RStudio* v. 1.3.1093 using the package *adegenet* (Jombart 2008; Jombart and Ahmed 2011), and with *STRUCTURE* v. 2.3.4 (Pritchard et al. 2000; Falush et al. 2003) implemented via the wrapper program *structure_threader* v. 1.3.10 (Pina-Martins et al. 2017). For *DAPC* analyses, which have been shown to be robust to the inclusion of singletons (Linck and Battey 2019), data sets not filtered for minimum minor allele count were used. For *STRUCTURE*, which has been shown to be sensitive to the inclusion of singletons (Linck and Battey 2019), data sets filtered for a minimum minor allele count of 3 were used. The following K-values were tested in *STRUCTURE*: 1–6 (*C. gracilis* complete data set) 1–5 (*C. gracilis* no-Senegal and *R. megacantha* complete data sets), and 1–4 (*R. megacantha* no-Senegal data set). For each K-value for each data set, ten independent *STRUCTURE* runs were completed, each with 1,000,000 generations with the first 50,000 generations discarded as burn–in. For *DAPC* analyses, the most likely number of populations was determined using the Bayesian information criterion (BIC) and the Akaike information criteria (AIC) and the number of retained principal components was determined through ascore optimization. For *STRUCTURE*, the most likely number genetic bins (i.e., k–value) was determined using likelihood scores and the Evanno ΔK method (Evanno et al. 2005) implemented in *structureHarvester* v. A.2 (Earl and VonHoldt 2012).

Phylogenetic analysis of SNP data sets was completed using *RAxML* v 8.2.11 (Stamatakis 2014). The complete data set not filtered for minimum minor allele count was used for both species. For each data set, loci were first concatenated for each specimen, and invariant sites were removed using the *Python 3* script *raxml_ascbias* (ascbias.py; https://github.com/btmartin721/raxml_ascbias#raxml_ascbias) in *Python* v. 3.9.7 (Van Rossum and Drake 2009). For both analyses, a GTR+Γ model of nucleotide substitution was specified with the –*m ASC_GTRGAMMA* flag, and standard ascertainment bias correction was specified with the ––*asccorr = lewis* flag to account for omission of invariant sites. To yield better likelihood scores, use of the median (rather than the mean) for the discrete Γ model of rate heterogeneity was specified with the –*u* flag. As outgroups were not included in either *RAxML* analysis, the resulting most likely topologies were rooted to maximize subtree balance. Nodal support was assessed with 1,000 rapid bootstrap replicates via the –*f a* and –*# 1000* flags. Bootstrap values (BS) were displayed on the most likely tree topology using *SumTrees* v. 4.5.2 (Sukumaran, J. and M. T. Holder. SumTrees: Phylogenetic Tree Summarization. 4.5.2. Available at https://github.com/jeetsukumaran/DendroPy) implemented in *DendroPy* v. 4.5.2 (Sukumaran and Holder 2010).

The *populations* module of *Stacks* was used to generate population-level summary statistics and corrected AMOVA F_ST_ values separately for both species using the complete data sets not filtered for minimum minor allele count. To assess whether genetic diversity is structured by geography or definitive host species, two iterations of *populations* were run for both species specifying different subpopulation groupings. The iterations run for *R. megacantha* were 1) specifying the specimens from Senegal, the specimens from Belize, and the specimens from South Carolina and the Gulf of Mexico as three separate populations and 2) specifying the specimens from *R*. *brasiliensis, R. bonasus*, and *R. marginata* as three separate populations. The iterations run for *C. gracilis* were 1) specifying six separate populations for the specimens from Senegal, Australia, South Carolina, Florida Atlantic, the Gulf of Mexico, and the northern Atlantic Ocean, and 2) specifying five separate populations for the specimens hosted by *C*. *brevipinna, C. limbatus*, *C.* cf. *limbatus, C. isodon*, and *R*. *terraenovae*.

To visualize levels of genetic divergence between conspecific specimens within and between infrapopulations, pairwise distances were calculated and plotted separately for both species using the package *adegenet* in *R* v. 4.0.3 via *RStudio* v. 1.3.1093. For each species, distances were calculated separately for both the complete and no-Senegal data sets not filtered for minimum minor allele count. Comparisons graphed include distances between members of the same infrapopulation for all infrapopulations where SNP data for more than a one specimen was available, and distances between members of each unique pair of infrapopulations.

## Supplementary material

Supplementary data are available at *Genome Biology and Evolution* online (http://www.gbe.oxfordjournals.org/).

## Supplementary Material

evad190_Supplementary_Data

## Data Availability

The data underlying this article are available in the Dryad Digital Repository at DOI 10.5061/dryad.q573n5tng.
