# Supplementary material for: First Insights into Population Structure and Genetic Diversity Versus Host Specificity in Trypanorhynch Tapeworms Using Multiplexed Shotgun Genotyping
Source: Genome Biol Evol. 2023 Oct 31;15(10):evad190. doi: 10.1093/gbe/evad190 (PMC10616631; doi:10.1093/gbe/evad190)
Supplement: evad190_Supplementary_Data [file evad190_supplementary_data.pdf]

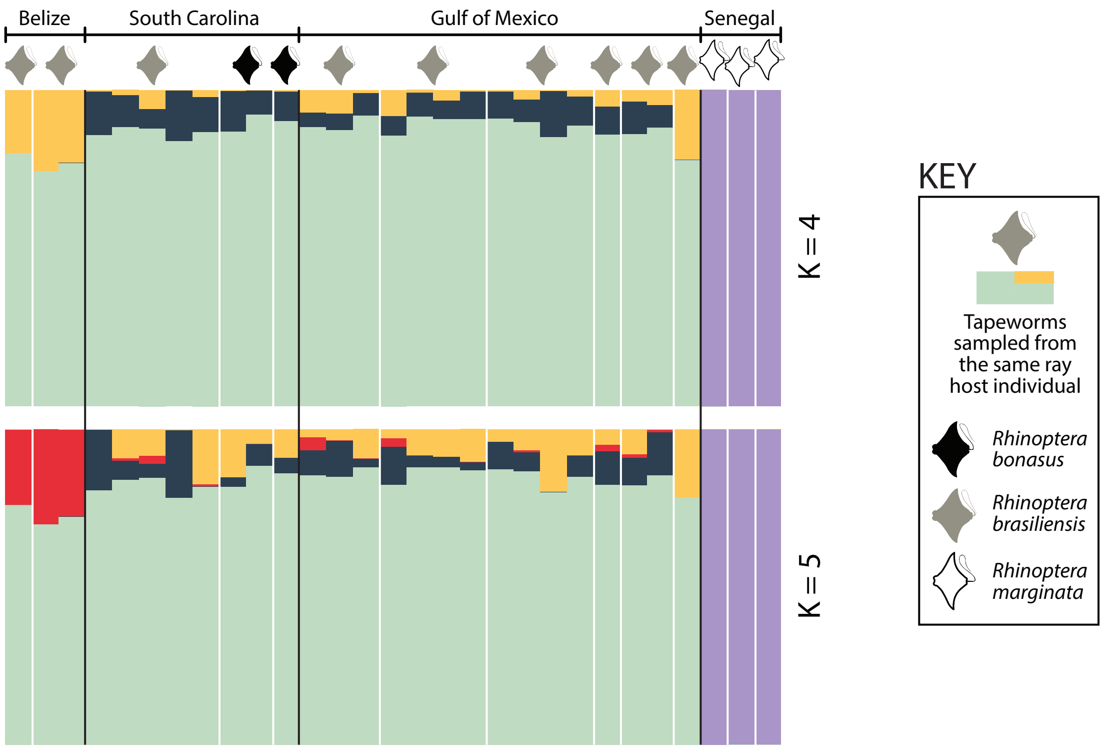

Supplementary Figure S1. Alternate, less common binning patterns produced by *STRUCTURE* for K-values of 4 and 5 for *Rhinoptericola megacantha* based on the complete single nucleotide polymorphism dataset. Black lines separate sampling localities and white lines separate host individuals within a sampling locality.

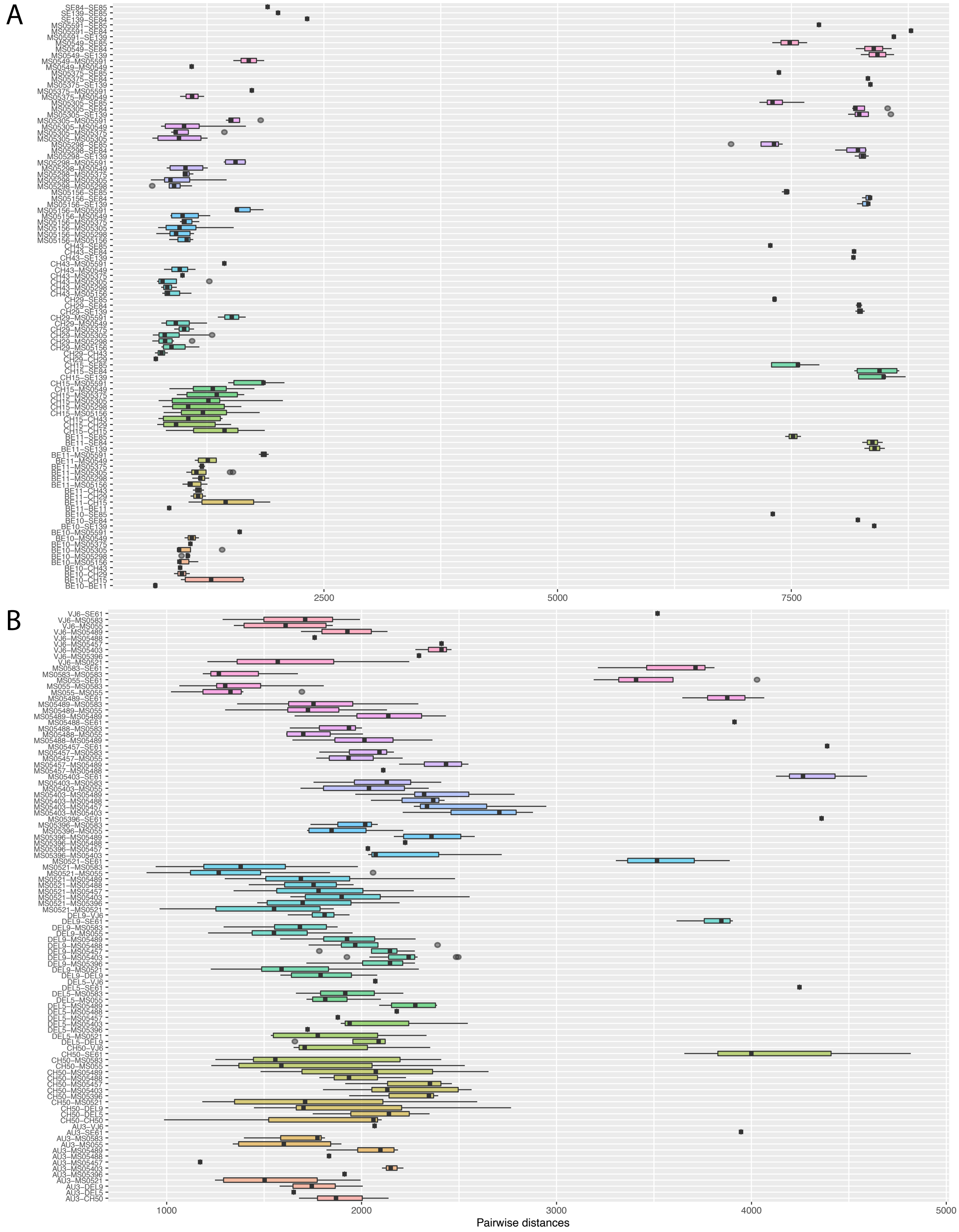

Supplementary Figure S2. Plots of pairwise distances between tapeworm specimens within an infracommunity and between pairs of infracommunities for *Rhinoptericola megacantha* (A) and *Callitetrarhynchus gracilis* (B) based on complete single nucleotide polymorphism datasets.

Supplementary Table S1. Size, sex, and detailed collection locality data for elasmobranch specimens hosting specimens of *Rhinoptericola megacantha* and *Callitetrarhynchus gracilis* for which sequence data were generated as part of this study. Asterisks (*) indicate an identification not verified with NADH2 sequence data; double asterisks (**) indicate a host name sensu Naylor et al. (2012).

Family: Species	Host accession number	Disk width/ total length (cm)	Sex	Collection date	Collection locality	No. worms sequenced: No. worms retained in final SNP datasets
Rhinoptericola megacantha						
Rhinopteridae: *Rhinoptera brasiliensis*	BE-10	89 DW	M	May 18, 2012	Gales Point Manatee (17°13'1.0"N, 88°19'01.4"W), Belize, Inner Channel, Atlantic Ocean	1:1
Rhinopteridae: *Rhinoptera brasiliensis*	BE-11	88 DW	F	May 18, 2012	Gales Point Manatee (17°13'1.0"N, 88°19'01.4"W), Belize, Inner Channel, Atlantic Ocean	4:2
Rhinopteridae: *Rhinoptera bonasus*	CH-3	88 DW	F	Jun. 27, 2012	Awendaw (33°02'07.78"N, 79°32'47.24"W), South Carolina, USA, Bulls Bay, Atlantic Ocean	1:0
Rhinopteridae: *Rhinoptera brasiliensis*	CH-15	58 DW	M	Jun. 17, 2013	Awendaw (33°0'34.27"N, 79°29'8.82"W), South Carolina, USA, Bulls Bay, 5 Fathom Creek, Atlantic Ocean	5:5
Rhinopteridae: *Rhinoptera bonasus*	CH-17	82.5 DW	M	Jun.17, 2013	Charleston (32°45'2.53"N, 79°53'48.28"W), South Carolina, USA, Charleston Harbor, Atlantic Ocean	1:0
Rhinopteridae: *Rhinoptera bonasus*	CH-29	87 DW	F	Jun. 19, 2013	Awendaw (33°02'07.78"N, 79°32'47.24"W), South Carolina, USA, Bulls Bay, Atlantic Ocean	2:2
Rhinopteridae: *Rhinoptera bonasus*	CH-30	93 DW	F	Jun. 19, 2013	Awendaw (33°02'07.78"N, 79°32'47.24"W), South Carolina, USA, Bulls Bay, Atlantic Ocean	1:0
Rhinopteridae: *Rhinoptera bonasus*	CH-43	94 DW	F	Jun. 15, 2015	Charleston, South Carolina, USA, Atlantic Ocean	1:1
Rhinopteridae: *Rhinoptera brasiliensis*	MS05-49	92 DW	M	Jun. 19, 2005	South side of East Ship Island (30°14'24.54"N, 88°52'25.25"W), Mississippi, USA, Gulf of Mexico	2:2
Rhinopteridae: *Rhinoptera brasiliensis**	MS05-156	?	?	Aug. 2005	Ship Island (30°13'13.53"N, 88°54'52.48"W), Mississippi, USA, Gulf of Mexico	3:3
Rhinopteridae: *Rhinoptera brasiliensis*	MS05-298	97 DW	F	Apr. 25, 2006	West tip of Horn Island (30°14'24.54"N, 88°52'25.25"W), Mississippi, USA, Gulf of Mexico	5:4
Rhinopteridae: *Rhinoptera brasiliensis**	MS05-305	81 DW	F	Mar. 28, 2006	Horn Island (30°15′04″N, 88°42′42″W), Mississippi, USA, Gulf of Mexico	5:4
Rhinopteridae: *Rhinoptera brasiliensis*	MS05-375	?	?	Aug. 27, 2006	West of south tip of Chandeleur Islands (29°57'9.54"N, 88°50'38.98"W), Louisiana, USA, Gulf of Mexico	1:1
Rhinopteridae: *Rhinoptera brasiliensis**	MS05-591	101.5 DW	M	Jun. 7, 2009	Horn Island (30°14'1.44"N, 88°40'5.47"W), Mississippi, USA, Gulf of Mexico	1:1
Rhinopteridae: *Rhinoptera marginata*	SE-78	54.5 DW	F	Jan. 12, 2003	St. Louis (16°1'28"N, 16°30'33"W), Senegal, Atlantic Ocean	1:0
Rhinopteridae: *Rhinoptera marginata*	SE-84	74 DW	F	Jan. 13, 2003	St. Louis (16°1'28"N, 16°30'33"W), Senegal, Atlantic Ocean	2:1
Rhinopteridae: *Rhinoptera marginata*	SE-85	56 DW	F	Jan. 13, 2003	St. Louis (16°1'28"N, 16°30'33"W), Senegal, Atlantic Ocean	1:1
Rhinopteridae: *Rhinoptera marginata*	SE-138	84.5 DW	F	Jan. 3, 2004	St. Louis (16°1'28"N, 16°30'33"W), Senegal, Atlantic Ocean	1:0
Rhinopteridae: *Rhinoptera marginata*	SE-139	86 DW	F	Jan. 3, 2004	St. Louis (16°1'28"N, 16°30'33"W), Senegal, Atlantic Ocean	1:1

Callitetrarhynchus gracilis						
Carcharhinidae: *Carcharhinus* cf. *limbatus***	AU-3	204 TL	F	Aug. 4, 1997	Darwin (12°20'11"S, 130°54'39"E), Northern Territory, Australia, Buffalo Creek, Timor Sea, Indian Ocean	1:1
Carcharhinidae: *Rhizoprionodon terraenovae*	CH-8	93.5 TL	M	Jun. 28, 2012	Wadmalaw Point (32°37'45.84"N, 80°16'02.26"W), South Carolina, USA, North Edisto River, Atlantic Ocean	3:0
Carcharhinidae: *Carcharhinus limbatus*	CH-50	149 TL	F	Jun. 18, 2015	Charleston, South Carolina, USA, Bulls Bay, Atlantic Ocean	3:3
Carcharhinidae: *Rhizoprionodon terraenovae*	DEL-5	67.5 TL	M	Apr. 18, 2001	Florida (24°47.56'N , 80°39.79'W), USA, Atlantic Ocean	1:1
Carcharhinidae: *Carcharhinus limbatus*	DEL-9	154.6 TL	M	Apr. 22, 2001	Florida, (28°0.8'N , 80°27'W), USA, Atlantic Ocean	5:4
Carcharhinidae: *Carcharhinus brevipinna*	MS05-5	103 TL	F	Jun. 15, 2005	South of the Shallow Fields (29°37'22.8"N, 88°30'11"W), Mississippi, USA, Gulf of Mexico	5:4
Carcharhinidae: *Rhizoprionodon terraenovae*	MS05-21	88 TL	M	Jun. 16, 2005	North of West end of Horn Island, (30°14'37.70"N, 88°46'37.62"W) Mississippi, USA, Gulf of Mexico	3:3
Carcharhinidae: *Carcharhinus limbatus*	MS05-24	80 TL	M	Jun. 16, 2005	North of West end of Horn Island, (30°14'37.70"N, 88°46'37.62"W) Mississippi, USA, Gulf of Mexico	1:1
Carcharhinidae: *Carcharhinus isodon*	MS05-83	82 TL	M	Jun. 21, 2005	Round Island (30°17'42.45"N, 88°35'11.55"W), Mississippi, USA, Gulf of Mexico	3:3
Carcharhinidae: *Rhizoprionodon terraenovae*	MS05-396	86 TL	M	Oct. 3, 2006	Florida (29°46'3"N, 85°21'4"W), USA, St. Joe's Bay, Gulf of Mexico	2:1
Carcharhinidae: *Carcharhinus limbatus*	MS05-403	100 TL	M	Oct. 4, 2006	Florida (29°59'37"N, 85°31'48"W), USA, Crooked Island Bay, Gulf of Mexico	5:3
Carcharhinidae: *Carcharhinus brevipinna**	MS05-457	79 TL	M	Oct. 8, 2006	Southwest end of Horn Island (30°14'9"N, 88°46'2"W), Mississippi, USA, Gulf of Mexico	1:1
Carcharhinidae: *Carcharhinus limbatus**	MS05-488	78 TL	F	May 22, 2007	Florida (29°40'8.05"N, 85°13'30.17"W), USA, Indian Pass, Gulf of Mexico	4:1
Carcharhinidae: *Carcharhinus limbatus**	MS05-489	79 TL	M	May 22, 2007	Florida (29°40'8.05"N, 85°13'30.17"W), USA, Indian Pass, Gulf of Mexico	5:4
Carcharhinidae: *Rhizoprionodon terraenovae**	VJ-6	94.4 TL	F	Jan. 25, 1997	USA, Atlantic Ocean	1:1
Carcharhinidae: *Carcharhinus brevipinna*	SE-61	71.5 TL	M	Jan. 11, 2003	Ouakam (14°42'54"N, 17°29'28"W), Senegal, Atlantic Ocean	2:1
Carcharhinidae: *Carcharhinus brevipinna*	SE-81	86 TL	F	Jan. 12, 2003	St. Louis (16°1'28"N, 16°30'33"W), Senegal, Atlantic Ocean	2:0

Abbreviations: DW–disk width; F–female; M–male; TL–total length.

Supplementary Table 2. Specimens sequenced for Illumina next generation sequencing (NGS) following multiplexed shotgun genotyping (MSG) library preparation and/or for Sanger sequencing of partial 28S rRNA (D1–D3 gene regions). Single asterisk (*) indicates that specimen underwent two rounds of MSG library preparation and NGS; double asterisks (**) indicate read counts following demultiplexing and initial quality filtering with *Stacks process_radtags* and *Trimmomatic*; dagger (†) indicates that a specimen extracted by colleagues at the University of Connecticut; double dagger (‡) indicates a host name sensu Naylor et al. (2012).

Species	Unique lab specimen no.	No. reads retained**	Reason for removal from final MSG datasets	Hologenophore accession no.	Host species	Unique host accession no.	Sampling locality
Rhinoptericola megacantha							
	KW399†	1,120,666		LRP 10432	*Rhinoptera brasiliensis*	BE-10	Belize
	BE-11-1*	707,855	SNPfiltR (DeRaad 2022)	LRP 10837	*Rhinoptera brasiliensis*	BE-11	Belize
	BE-11-2*	869,764	SNPfiltR (DeRaad 2022)	LRP 10838	*Rhinoptera brasiliensis*	BE-11	Belize
	BE-11-3	9,444,812		LRP 10433	*Rhinoptera brasiliensis*	BE-11	Belize
	BE-11-4	1,283,042		LRP 10839	*Rhinoptera brasiliensis*	BE-11	Belize
	CH-3-1*	1,030,419	SNPfiltR (DeRaad 2022)	LRP 10440	*Rhinoptera bonasus*	CH-3	South Carolina
	CH-15-1*	3,156,060		LRP 10434	*Rhinoptera brasiliensis*	CH-15	South Carolina
	CH-15-3	4,191,960		LRP 10840	*Rhinoptera brasiliensis*	CH-15	South Carolina
	CH-15-4	4,672,947		LRP 10435	*Rhinoptera brasiliensis*	CH-15	South Carolina
	CH-15-5*	3,611,116		LRP 10436	*Rhinoptera brasiliensis*	CH-15	South Carolina
	CH-15-6	1,534,189		LRP 10841	*Rhinoptera brasiliensis*	CH-15	South Carolina
	CH-17-1*	126,479	SNPfiltR (DeRaad 2022)	LRP 10437	*Rhinoptera bonasus*	CH-17	South Carolina
	CH-29-1	2,349,107		LRP 10439	*Rhinoptera bonasus*	CH-29	South Carolina
	CH-29-2	1,435,022		LRP 10842	*Rhinoptera bonasus*	CH-29	South Carolina
	CH-30-1*	154,402	SNPfiltR (DeRaad 2022)	LRP 10441	*Rhinoptera bonasus*	CH-30	South Carolina
	CH-43-1	1,412,893		LRP 10843	*Rhinoptera bonasus*	CH-43	South Carolina
	MS05-49-1	5,569,289		LRP 10844	*Rhinoptera brasiliensis*	MS05-49	Gulf of Mexico
	MS05-49-2*	1,433,907		LRP 10450	*Rhinoptera brasiliensis*	MS05-49	Gulf of Mexico
	MS05-156-1	1,733,092		LRP 10442	*Rhinoptera brasiliensis*	MS05-156	Gulf of Mexico
	MS05-156-2*	5,495,368		LRP 10443	*Rhinoptera brasiliensis*	MS05-156	Gulf of Mexico
	MS05-156-3	901,702		LRP 10845	*Rhinoptera brasiliensis*	MS05-156	Gulf of Mexico
	MS05-298-20	5,558,729		LRP 10444	*Rhinoptera brasiliensis*	MS05-298	Gulf of Mexico
	MS05-298-21	1,219,614		LRP 10846	*Rhinoptera brasiliensis*	MS05-298	Gulf of Mexico
	MS05-298-22*	1,157,913		LRP 10445	*Rhinoptera brasiliensis*	MS05-298	Gulf of Mexico
	MS05-298-23*	930,097	SNPfiltR (DeRaad 2022)	LRP 10847	*Rhinoptera brasiliensis*	MS05-298	Gulf of Mexico
	MS05-298-24	920,720		LRP 10446	*Rhinoptera brasiliensis*	MS05-298	Gulf of Mexico
	MS05-305-1	1,540,560		LRP 10848	*Rhinoptera brasiliensis*	MS05-305	Gulf of Mexico
	MS05-305-2*	115,747	SNPfiltR (DeRaad 2022)	LRP 10849	*Rhinoptera brasiliensis*	MS05-305	Gulf of Mexico
	MS05-305-3	1,889,779		LRP 10448	*Rhinoptera brasiliensis*	MS05-305	Gulf of Mexico
	MS05-305-4*	8,422,210		LRP 10447	*Rhinoptera brasiliensis*	MS05-305	Gulf of Mexico
	MS05-305-5	1,281,232		LRP 10850	*Rhinoptera brasiliensis*	MS05-305	Gulf of Mexico
	MS05-375-1	13,950,789		LRP 10449	*Rhinoptera brasiliensis*	MS05-375	Gulf of Mexico
	MS05-591-1*	1,803,326		LRP 10851	*Rhinoptera brasiliensis*	MS05-591	Gulf of Mexico
	SE-78-1*	541,933	SNPfiltR (DeRaad 2022)	LRP 10852	*Rhinoptera marginata*	SE-78	Senegal
	SE-84-1*	749,650	SNPfiltR (DeRaad 2022)	LRP 10452	*Rhinoptera marginata*	SE-84	Senegal
	JW555†	21,645,555		LRP 10853	*Rhinoptera marginata*	SE-84	Senegal
	SE-85-1	1,164,486		LRP 10854	*Rhinoptera marginata*	SE-85	Senegal

	SE-138-1*	30,263	SNPfiltR (DeRaad 2022)	LRP 10855	*Rhinoptera marginata*	SE-138	Senegal
	SE-139-1*	10,527,402		LRP 10451	*Rhinoptera marginata*	SE-139	Senegal
Callitetrarhynchus gracilis							
	AU-3-1	5,630,185		LRP 10856	*Carcharhinus* cf. *limbatus*‡	AU-3	N. Territory, Australia
	CH-8-1*	1,304,745	bad apple (Cerca et al. 2021)	LRP 10857	*Rhizoprionodon terraenovae*	CH-8	South Carolina
	CH-8-2*	1,411,674	bad apple (Cerca et al. 2021)	LRP 10858	*Rhizoprionodon terraenovae*	CH-8	South Carolina
	CH-8-3*	1,242,686	bad apple (Cerca et al. 2021)	LRP 10859	*Rhizoprionodon terraenovae*	CH-8	South Carolina
	CH-50-1	8,070,627		LRP 10860	*Carcharhinus limbatus*	CH-50	South Carolina
	CH-50-2*	18,035,193		LRP 10861	*Carcharhinus limbatus*	CH-50	South Carolina
	KW840†	15,303,104		LRP 10862	*Carcharhinus limbatus*	CH-50	South Carolina
	JW474†	25,058,762		LRP 10863	*Rhizoprionodon terraenovae*	DEL-5	Atlantic Florida
	DEL-9-1*	38,171,518		LRP 10864	*Carcharhinus limbatus*	DEL-9	Atlantic Florida
	DEL-9-2	19,922,341		LRP 10865	*Carcharhinus limbatus*	DEL-9	Atlantic Florida
	DEL-9-3*	7,735,470		LRP 10866	*Carcharhinus limbatus*	DEL-9	Atlantic Florida
	DEL-9-4*	938,324	bad apple (Cerca et al. 2021)	LRP 10867	*Carcharhinus limbatus*	DEL-9	Atlantic Florida
	DEL-9-5*	4,091,167		LRP 10868	*Carcharhinus limbatus*	DEL-9	Atlantic Florida
	MS05-5-1*	2,674,933	SNPfiltR (DeRaad 2022)	LRP 10869	*Carcharhinus brevipinna*	MS05-5	Gulf of Mexico
	MS05-5-2	41,506,914		LRP 10870	*Carcharhinus brevipinna*	MS05-5	Gulf of Mexico
	MS05-5-3	3,272,653		LRP 10871	*Carcharhinus brevipinna*	MS05-5	Gulf of Mexico
	MS05-5-4	4,454,885		LRP 10872	*Carcharhinus brevipinna*	MS05-5	Gulf of Mexico
	MS05-5-5	7,926,344		LRP 10873	*Carcharhinus brevipinna*	MS05-5	Gulf of Mexico
	MS05-21-2*	3,522,108		LRP 10874	*Rhizoprionodon terraenovae*	MS05-21	Gulf of Mexico
	MS05-21-3*	5,110,408		LRP 10875	*Rhizoprionodon terraenovae*	MS05-21	Gulf of Mexico
	MS05-21-4	5,192,806		LRP 10876	*Rhizoprionodon terraenovae*	MS05-21	Gulf of Mexico
	MS05-24-1	4,201,041		LRP 10877	*Carcharhinus limbatus*	MS05-24	Gulf of Mexico
	MS05-83-1	3,612,175		LRP 10878	*Carcharhinus isodon*	MS05-83	Gulf of Mexico
	MS05-83-2	8,573,745		LRP 10879	*Carcharhinus isodon*	MS05-83	Gulf of Mexico
	MS05-83-3*	7,970,821		LRP 10880	*Carcharhinus isodon*	MS05-83	Gulf of Mexico
	MS05-396-1*	36,062,437		LRP 10881	*Rhizoprionodon terraenovae*	MS05-396	Gulf of Mexico
	JW477*†	4,522,503	bad apple (Cerca et al. 2021)	LRP 10882	*Rhizoprionodon terraenovae*	MS05-396	Gulf of Mexico
	MS05-403-1*	2,867,690	bad apple (Cerca et al. 2021)	LRP 10883	*Carcharhinus limbatus*	MS05-403	Gulf of Mexico
	MS05-403-2*	1,242,006	SNPfiltR (DeRaad 2022)	LRP 10884	*Carcharhinus limbatus*	MS05-403	Gulf of Mexico
	MS05-403-3*	20,996,906		LRP 10885	*Carcharhinus limbatus*	MS05-403	Gulf of Mexico
	MS05-403-4*	21,070,495		LRP 10886	*Carcharhinus limbatus*	MS05-403	Gulf of Mexico
	MS05-403-5*	14,023,118		LRP 10887	*Carcharhinus limbatus*	MS05-403	Gulf of Mexico
	MS05-457-1*	4,667,177		LRP 10888	*Carcharhinus brevipinna*	MS05-457	Gulf of Mexico
	MS05-488-1*	2,941,599	bad apple (Cerca et al. 2021)	LRP 10889	*Carcharhinus limbatus*	MS05-488	Gulf of Mexico
	MS05-488-2*	4,035,585	bad apple (Cerca et al. 2021)	LRP 10890	*Carcharhinus limbatus*	MS05-488	Gulf of Mexico
	MS05-488-3*	10,186,793		LRP 10891	*Carcharhinus limbatus*	MS05-488	Gulf of Mexico
	MS05-488-4*	1,229,041	bad apple (Cerca et al. 2021)	LRP 10892	*Carcharhinus limbatus*	MS05-488	Gulf of Mexico
	MS05-489-3*	11,808,355		LRP 10893	*Carcharhinus limbatus*	MS05-489	Gulf of Mexico
	MS05-489-4	5,020,369		LRP 10894	*Carcharhinus limbatus*	MS05-489	Gulf of Mexico
	MS05-489-5*	15,639,315		LRP 10895	*Carcharhinus limbatus*	MS05-489	Gulf of Mexico
	MS05-489-6*	20,044,128		LRP 10896	*Carcharhinus limbatus*	MS05-489	Gulf of Mexico
	MS05-489-7*	5,327,316	bad apple (Cerca et al. 2021)	LRP 10897	*Carcharhinus limbatus*	MS05-489	Gulf of Mexico
	VJ-6-1	5,028,827		LRP 10898	*Rhizoprionodon terraenovae*	VJ-6	N. Atlantic Ocean
	SE-61-1	1,698,962	SNPfiltR (DeRaad 2022)	LRP 10899	*Carcharhinus brevipinna*	SE-61	Senegal
	SE-61-2*	7,562,834		LRP 10900	*Carcharhinus brevipinna*	SE-61	Senegal

SE-81-1*	12,002,596	bad apple (Cerca et al. 2021)	LRP 10901	*Carcharhinus brevipinna*	SE-81	Senegal
SE-81-2-HUP*	18,338	SNPfiltR (DeRaad 2022)	LRP 10902	*Carcharhinus brevipinna*	SE-81	Senegal

References

Cerca J, et al. 2021. Removing the bad apples: A simple bioinformatic method to improve loci-recovery in de novo RADseq data for non-model organisms. Methods Ecol. Evol. 12: 805–817. doi: 10.1111/2041-210X.13562

DeRaad D. 2022. SNPfiltR: Interactively filter SNP datasets. Version R package version 0.1.1.

Naylor GJ, et al. 2012. A DNA sequence-based approach to the identification of shark and ray species and its implications for global elasmobranch diversity and parasitology. Bull. Am. Mus. Nat. Hist. 367: 262 pp. doi: 10.1206/754.1

Supplementary Table S3. Known definitive host associations of *Rhinoptericola megacantha* and *Callitetrarhynchus gracilis* with updated host identifications. Bolded host names indicate host species that were examined as part of this study, but from which specimens of *C. gracilis* were not recovered; asterisks (*) indicate a host name sensu Naylor et al. (2012); double asterisks (**) indicate a report from freshwater; daggers (†) indicate a report as "*Callitetrarhynchus* cf. *gracilis*".

Host order: Host family	Host species	Locality	Source of report	New report herein	Included herein
Rhinoptericola megacantha					
Myliobatiformes: Rhinopteridae	*Rhinoptera bonasus*	Chesapeake Bay, VA, USA	Carval and Campbell (1975)		
		Charleston, SC, USA	Herzog and Jensen (2022); this study		★
Myliobatiformes: Rhinopteridae	*Rhinoptera bonasus* or *Rhinoptera brasiliensis* (as *Rhinoptera bonasus*)	Gulf of Venezuela	Mayes and Brooks (1981)		
Myliobatiformes: Rhinopteridae	*Rhinoptera brasiliensis*	Charleston, SC, USA	Herzog and Jensen (2022); this study		★
Myliobatiformes: Rhinopteridae	*Rhinoptera brasiliensis* (as *Rhinoptera bonasus* prior to Herzog and Jensen, 2022)	Gulf of Mexico	Palm et al. (2009); Olson et al. (2010); Herzog and Jensen (2022); this study		★
Myliobatiformes: Rhinopteridae	*Rhinoptera brasiliensis*	Belize	Herzog and Jensen (2022); this study		★
		S. and S.E. Brazil	Napoleão et al. (2015)		
Myliobatiformes: Rhinopteridae	*Rhinoptera marginata*	Senegal	Herzog and Jensen (2022); this study		★
Myliobatiformes: Dasyatidae	*Hypanus say*	Charleston, SC, USA	Herzog and Jensen (2022)		
Callitetrarhynchus gracilis					
Carcharhiniformes: Carcharhinidae	***Carcharhinus amblyrhynchoides***	Queensland, Australia	Palm (2004)		
Carcharhiniformes: Carcharhinidae	***Carcharhinus amboinensis***	N. Territory, Australia	Palm (2004); Olson et al. (2010)		
Carcharhiniformes: Carcharhinidae	*Carcharhinus brevipinna*	Gulf of Mexico	This study	★	★
		Senegal	This study	★	★
Carcharhiniformes: Carcharhinidae	*Carcharhinus* cf. *dussumieri**	Iran, Persian Gulf	Haseli et al. (2010)		
Carcharhiniformes: Carcharhinidae	*Carcharhinus fitzroyensis*	N. Territory, Australia	Palm (2004)		
Carcharhiniformes: Carcharhinidae	*Carcharhinus isodon*	Gulf of Mexico	This study	★	★
Carcharhiniformes: Carcharhinidae	***Carcharhinus leucas***	Costa Rica	Watson and Thorson (1976)**		
		Gulf of Mexico	Palm (2004); Méndez and González (2013)		
Carcharhiniformes: Carcharhinidae	*Carcharhinus limbatus*	Charleston, SC, USA	This study	★	★
		Florida, USA	This study	★	★
		Gulf of Mexico	Palm and Overstreet (2000); Owens (2008); this study		★
Carcharhiniformes: Carcharhinidae	*Carcharhinus* cf. *limbatus**	N. Territory, Australia	This study	★	★
Carcharhiniformes: Carcharhinidae	*Carcharhinus melanopterus*	Queensland, Australia	Olson et al. (2001)		
Carcharhiniformes: Carcharhinidae	***Carcharhinus obscurus***	Japan	Nakajima and Egusa (1972a)		
Carcharhiniformes: Carcharhinidae	***Carcharhinus sorrah*** (as *Carcharhinus* cf. *sorrah)*	Iran, Persian Gulf	Haseli et al. (2010)		

Carcharhiniformes: Carcharhinidae	***Lamiopsis tephrodes***	Borneo	Schaeffner and Beveridge (2014)		
Carcharhiniformes: Carcharhinidae	*Negaprion brevirostris*	Japan	Nakajima and Egusa (1972a)		
Carcharhiniformes: Carcharhinidae	***Prionace glauca***	California, USA	Heinz and Dailey (1974)		
Carcharhiniformes: Carcharhinidae	*Rhizoprionodon* cf. *acutus* 2* (as *Rhizoprionodon acutus*)	Japan	Nakajima and Egusa (1972a)		
Carcharhiniformes: Carcharhinidae	***Rhizoprionodon* cf. *acutus* 1*** (as *Rhizoprionodon acutus*)	South Africa	Palm (2004)		
Carcharhiniformes: Carcharhinidae	*Rhizoprionodon acutus*	Iran, Persian Gulf	Haseli et al. (2010)		
Carcharhiniformes: Carcharhinidae	***Rhizoprionodon* cf. *acutus* 3*** (as *Rhizoprionodon acutus* 3)	Borneo	Schaeffner and Beveridge (2014)		
Carcharhiniformes: Carcharhinidae	*Rhizoprionodon terraenovae*	Charleston, SC, USA	This study	★	★
		Florida, USA	This study	★	★
		Gulf of Mexico	Palm (2004); this study		★
		Senegal	Palm (2004)		
Carcharhiniformes: Sphyrnidae	***Sphyrna lewini* 1*** (as *Sphyrna lewini*)	Gulf of Mexico	Palm (1995)		
Carcharhiniformes: Sphyrnidae	***Sphyrna zygaena***	Japan	Nakajima and Egusa (1972b)		
Carcharhiniformes: Triakidae	***Mustelus canis***	Rio Grande do Sul, Brazil	São Clemente and Gomes (1989)		
Carcharhiniformes: Triakidae	*Mustelus moisis*	Iraq, Persian Gulf	Mhaisen et al. (2018)†		
Carcharhiniformes: Triakidae	*Triakis scyllium*	Japan	Nakajima and Egusa (1972c); Nakajima and Egusa (1973)		
Orectolobiformes: Ginglymostomatidae	***Nebrius ferrugineus***	Sri Lanka	Beveridge and Campbell (1998)		
Myliobatiformes: Dasyatidae	*Hemitrygon fluviorum* (as *Dasyatis fluviorum*)	Queensland, Australia	Palm (2004)		

REFERENCES

Beveridge I, Campbell R. 1998. Re-examination of the trypanorhynch cestode collections of A.E. Shipley, J. Hornell and T. Southwell, with the erection of a new genus, *Trygonicola*, and redescriptions of seven species. Syst. Parasitol. 39: 1–34. doi: 10.1023/A:1005852507995

Carvajal J, Campbell RA. 1975. *Rhinoptericola megacantha* gen. et sp. n., representing a new family of trypanorhynch cestodes from the cownose ray, *Rhinoptera bonasus* (Mitchill 1815). J. Parasitol. 61: 1023–1030. doi: 10.2307/3279368

Haseli M, Malek M, Palm HW. 2010. Trypanorhynch cestodes of elasmobranchs from the Persian Gulf. Zootaxa 2492: 28–48. doi: 10.11646/zootaxa.2492.1.2

Heinz ML, Dailey MD. 1974. The Trypanorhyncha (Cestoda) of elasmobranch fishes from southern California and northern Mexico. Proc. Helminthol. Soc. Wash. 41: 161–169.

Herzog KS, Jensen K. 2022. A synergistic, global approach to revising the trypanorhynch tapeworm family Rhinoptericolidae (Trypanobatoida). PeerJ 10:e12865. doi: 10.7717/peerj.12865

Mayes MA, Brooks DR. 1981. Cestode parasites of some Venezuelan stingrays. Proc. Biol. Soc. Wash. 93: 1230–1238.

Méndez O, González MÁD. 2013. Cestodos del tiburón toro *Carcharhinus leucas* en playa Chachalacas, Veracruz, México. Neotrop. Helminthol. 7: 167–171. doi: 10.24039/rnh201371959

Mhaisen FT, Ali AH, Khamees NR. 2018. Marine fish parasitology of Iraq: A review and checklists. Biol. Appl. Environ. Res. 2: 231–297.

Nakajima K, Egusa S. 1972a. [Studies on a new trypanorhynchan larvae, *Callitetrarhynchus* sp., parasitic in cultured yellowtail-IX. Final host] (in Japanese; English abstract). Fish Pathol. 6: 78–82. doi: 10.3147/jsfp.6.78

Nakajima K, Egusa S. 1972b. [Studies on a new trypanorhynchan larva, *Callitetrarhynchus* sp., parasitic on cultured yellowtail-XV. Life cycle] (in Japanese; English abstract). Fish Pathol. 7: 6–14. doi: 10.3147/jsfp.7.6

Nakajima K, Egusa S. 1972c. [Studies on a new trypanorhynchan larvae, *Callitetrarhynchus* sp., parasitic in cultured yellowtail-XI. Growth of the adult in the spiral valve of *Triakis scyllia*] (in Japanese; English abstract). Bull. Japan. Soc. Sci. Fish. 38: 945–954. doi: 10.2331/suisan.38.945

Nakajima K, Egusa S. 1973. [Studies on a new trypanorhynchan larvae, *Callitetrarhynchus* sp., parasitic in cultured yellowtail-XIII. Morphology of the adult and its taxonomy] (in Japanese; English abstract). Bull. Japan. Soc. Sci. Fish. 38: 149–158. doi: 10.2331/suisan.39.149

Napoleão S, Antonucci A, Amorim A, Takemoto R. 2015. Occurrence of *Rhinoptericola megacantha* (Cestoda, Trypanorhyncha) in new host and new location. Arq. Bras. Med. Vet. Zootec. 67: 1175–1177. doi: 10.1590/1678-4162-7015

Olson PD, et al. 2010. Evolution of the trypanorhynch tapeworms: Parasite phylogeny supports independent lineages of sharks and rays. Int. J. Parasitol. 40: 223–242. doi: 10.1016/j.ijpara.2009.07.012

Olson PD, Littlewood DTJ, Bray RA, Mariaux J. 2001. Interrelationships and evolution of the tapeworms (Platyhelminthes: Cestoda). Mol. Phylogenet. Evol. 19: 443–467. doi: 10.1006/mpev.2001.0930

Owens HL. 2008. Multiple species of *Phoreiobothrium* from the blacktip shark, *Carcharhinus limbatus*, in the Gulf of Mexico. Master’s thesis. University of Kansas, Lawrence, KS, USA.

Palm HW. 1995. Untersuchungen zur Systematik von Rüsselbandwürmern (Cestoda: Trypanorhyncha) aus atlantischen Fischen. Doctoral dissertation. Christian-Albrechts-Universität, Kiel, Germany. doi: 10.3289/IFM_BER_275

Palm HW. 2004. *The Trypanorhyncha Diesing, 1863*. Bogor, Indonesia: PKSPL-IPB Press.

Palm HW, Overstreet RM. 2000. New records of trypanorhynch cestodes from the Gulf of Mexico, including *Kotorella pronosoma* (Stossich, 1901) and *Heteonybelinia palliata* (Linton, 1924) comb. n. Folia Parasitol. 47: 293–302. doi: 10.14411/fp.2000.051

Palm HW, Waeschenbach A, Olson PD, Littlewood DTJ. 2009. Molecular phylogeny and evolution of the Trypanorhyncha (Platyhelminthes: Cestoda). Mol. Phylogenet. Evol. 52: 351–367. doi: 10.1016/j.ympev.2009.01.019

São Clemente SC, Gomes DC. 1989. Trypanorhyncha from sharks of southern Brazilian coast: *Eutetrarhynchus vooremi* sp. n. and two other species parasites of *Mustelus* (Pisces, Triakidae). Mem. Inst. Oswaldo Cruz 84: 475–481. doi: 10.1590/S0074-02761989000800083Schaeffner BC, Beveridge I. 2014. The trypanorhynch cestode fauna of Borneo. Zootaxa 3900: 021–049. doi: 10.11646/zootaxa.3900.1.2

Watson DE, Thorson TB. 1976. Helminths from elasmobranchs in Central American fresh waters. In: Thorson TB, editor. *Investigations of the Ichyofauna of Nicaraguan Lakes*. Lincoln, Nebrasksa, USA: School of Life Sciences, University of Nebraska-Lincoln. p. 629–640.

Supplementary Table S4. Host specimens examined for *Rhinoptericola megacantha* and *Callitetrarhynchus gracilis*. Asterisks (*) indicate host names sensu Naylor et al. (2012).

Host order: Host family	Host species	Collection locality	No. specimens examined	Focal species recovered
Carcharhiniformes: Carcharhinidae	*Carcharhinus acronotus*	Gulf of Mexico	1	
Carcharhiniformes: Carcharhinidae	*Carcharhinus amblyrhynchoides*	Borneo	1	
		India	2	
Carcharhiniformes: Carcharhinidae	*Carcharhinus amboinensis* 1*	Australia	1	
Carcharhiniformes: Carcharhinidae	*Carcharhinus brachyurus*	Korea	2	
Carcharhiniformes: Carcharhinidae	*Carcharhinus brevipinna*	Borneo	1	
		Gulf of Mexico	5	*Callitetrarhynchus gracilis*
		Senegal	4	*Callitetrarhynchus gracilis*
Carcharhiniformes: Carcharhinidae	*Carcharhinus* cf. *cautus**	Solomon Islands	1	
Carcharhiniformes: Carcharhinidae	*Carcharhinus coatesi*	Australia	2	
Carcharhiniformes: Carcharhinidae	*Carcharhinus falciformis*	Florida, USA	1	
Carcharhiniformes: Carcharhinidae	*Carcharhinus isodon*	Gulf of Mexico	4	*Callitetrarhynchus gracilis*
		South Carolina, USA	2	*Callitetrarhynchus gracilis*
Carcharhiniformes: Carcharhinidae	*Carcharhinus leucas*	Senegal	1	
Carcharhiniformes: Carcharhinidae	*Carcharhinus* cf. *leucas**	Borneo	1	
Carcharhiniformes: Carcharhinidae	*Carcharhinus limbatus*	Gulf of Mexico	7	*Callitetrarhynchus gracilis*
		Florida, USA	2	*Callitetrarhynchus gracilis*
		South Carolina, USA	3	*Callitetrarhynchus gracilis*
Carcharhiniformes: Carcharhinidae	*Carcharhinus* cf. *limbatus**	Australia	3	*Callitetrarhynchus gracilis*
		Sri Lanka	1	
Carcharhiniformes: Carcharhinidae	*Carcharhinus obscurus*	Florida, USA	1	
		Senegal	1	
Carcharhiniformes: Carcharhinidae	*Carcharhinus sealei*	Borneo	3	
Carcharhiniformes: Carcharhinidae	*Carcharhinus sorrah*	Borneo	3	
		Thailand	1	
		Taiwan	1	
		Viet Nam	1	
Carcharhiniformes: Carcharhinidae	*Carcharhinus* cf. *sorrah**	Australia	1	
Carcharhiniformes: Carcharhinidae	*Carcharhinus tilstoni*	Australia	1	
Carcharhiniformes: Carcharhinidae	*Lamiopsis tephrodes*	Borneo	4	
Carcharhiniformes: Carcharhinidae	*Loxodon* cf. *macrorhinus**	Mozambique	3	
Carcharhiniformes: Carcharhinidae	*Negaprion acutidens*	Australia	8	
Carcharhiniformes: Carcharhinidae	*Prionace glauca*	New York, USA	23	
Carcharhiniformes: Carcharhinidae	*Rhizoprionodon* cf. *acutus* 1*	Senegal	3	

Carcharhiniformes: Carcharhinidae	*Rhizoprionodon* cf. *acutus* 3*	Borneo	1	
Carcharhiniformes: Carcharhinidae	*Rhizoprionodon longurio*	Gulf of California	1	
Carcharhiniformes: Carcharhinidae	*Rhizoprionodon oligolinx*	Borneo	1	
		Sri Lanka	1	
Carcharhiniformes: Carcharhinidae	*Rhizoprionodon terraenovae*	Atlantic Ocean	1	*Callitetrarhynchus gracilis*
		Gulf of Mexico	6	*Callitetrarhynchus gracilis*
		Florida, USA	2	*Callitetrarhynchus gracilis*
		South Carolina, USA	1	*Callitetrarhynchus gracilis*
Carcharhiniformes: Carcharhinidae	*Scoliodon* cf. *lauticaudus**	India	1	
Carcharhiniformes: Carcharhinidae	*Scoliodon macrorhynchos*	Borneo	1	
		Taiwan	1	
		Viet Nam	4	
Carcharhiniformes: Carcharhinidae	*Triaenodon obesus*	Solomon Islands	2	
Carcharhiniformes: Galeocerdidae	*Galeocerdo* cf. *cuvier**	Gulf of Mexico	1	
Carcharhiniformes: Sphyrnidae	*Sphyrna lewini* 1*	Gulf of Mexico	3	
		Florida, USA	4	
Carcharhiniformes: Sphyrnidae	*Sphyrna lewini* 2*	Gulf of California	2	
		Taiwan	1	
Carcharhiniformes: Sphyrnidae	*Sphyrna* cf. *lewini**	Borneo	1	
Carcharhiniformes: Sphyrnidae	*Sphyrna mokarran* 1*	Gulf of Mexico	2	
		Florida, USA	1	
Carcharhiniformes: Sphyrnidae	*Sphyrna mokarran* 2*	Australia	3	
Carcharhiniformes: Sphyrnidae	*Sphyrna tiburo*	Gulf of Mexico	5	
		South Carolina, USA	4	
Carcharhiniformes: Sphyrnidae	*Sphyrna zygaena*	Gulf of California	1	
		Japan	3	
		Senegal	1	
		Taiwan	2	
Carcharhiniformes: Triakidae	*Galeorhinus galeus*	New Zealand	1	
Carcharhiniformes: Triakidae	*Mustelus canis*	Atlantic Ocean	1	
		Long Island Sound	4	
		Rhode Island, USA	2	
Carcharhiniformes: Triakidae	*Mustelus* cf. *antarcticus**	Solomon Islands	1	
Orectolobiformes: Ginglymostomatidae	*Nebrius ferrugineus*	Australia	4	
Myliobatiformes: Rhinopteridae	*Rhinoptera bonasus*	South Carolina, USA	5	*Rhinoptericola megacantha*
Myliobatiformes: Rhinopteridae	*Rhinoptera brasiliensis*	Belize	2	*Rhinoptericola megacantha*
		Gulf of Mexico	6	*Rhinoptericola megacantha*
		South Carolina, USA	1	*Rhinoptericola megacantha*
Myliobatiformes: Rhinopteridae	*Rhinoptera marginata*	Senegal	5	*Rhinoptericola megacantha*

Supplementary Table S5. Number of individuals, number of loci, and population genomic analyses conducted for each of the final filtered single nucleotide polymorphism datasets generated for *Rhinoptericola megacantha* and *Callitetrarhynchus gracilis*.

Species	Dataset	No. specimens	No. loci	Analyses conducted
Rhinoptericola megacantha				
	complete	29 (of 39)	2,568	*DAPC*; *RAxML*; summary statistics; pairwise distances
	no-Senegal	26 (of 34)	2,221	*DAPC*; pairwise distances
	MAC complete	29 (of 39)	1,958	*STRUCTURE*
	MAC no-Senegal	26 (of 34)	1,408	*STRUCTURE*
Callitetrarhynchus gracilis				
	complete	32 (of 47)	3,908	*DAPC*; *RAxML*; summary statistics; pairwise distances
	no-Senegal	31 (of 43)	3,273	*DAPC*; pairwise distances
	MAC complete	32 (of 47)	1,603	*STRUCTURE*
	MAC no-Senegal	31 (of 43)	1,294	*STRUCTURE*

Abbreviations: MAC–filtered for a minimum minor allele count of 3.

Supplementary Table S6. Pairwise distances between tapeworm specimens within an infracommunity and between pairs of infracommunities for *Rhinoptericola megacantha* and *Callitetrarhynchus gracilis* based on "no-Senegal" single nucleotide polymorphism datasets.

Species	Host of specimen 1	Host of specimen 2	Pairwise distance	Comparison made is within a single host individual (i.e., within an infracommunity)
Rm	BE11	BE11	1214.0267	TRUE
Rm	CH15	CH15	2011.693	TRUE
Rm	CH15	CH15	2043.3255	TRUE
Rm	CH15	CH15	2322.9347	TRUE
Rm	CH15	CH15	2042.1537	TRUE
Rm	CH15	CH15	1435.3626	TRUE
Rm	CH15	CH15	1734.0395	TRUE
Rm	CH15	CH15	1455.174	TRUE
Rm	CH15	CH15	1900.0789	TRUE
Rm	CH15	CH15	1433.2939	TRUE
Rm	CH15	CH15	1751	TRUE
Rm	CH29	CH29	1035.2497	TRUE
Rm	MS05156	MS05156	1449.7591	TRUE
Rm	MS05156	MS05156	1276.3753	TRUE
Rm	MS05156	MS05156	1551.2004	TRUE
Rm	MS05298	MS05298	1463.598	TRUE
Rm	MS05298	MS05298	1256.2964	TRUE
Rm	MS05298	MS05298	1549.635	TRUE
Rm	MS05298	MS05298	1115.3323	TRUE
Rm	MS05298	MS05298	1011.1408	TRUE
Rm	MS05298	MS05298	1214.4703	TRUE
Rm	MS05305	MS05305	1044.941	TRUE
Rm	MS05305	MS05305	1333.2847	TRUE
Rm	MS05305	MS05305	1079.8458	TRUE
Rm	MS05305	MS05305	1409.7987	TRUE
Rm	MS05305	MS05305	1173.9584	TRUE
Rm	MS05305	MS05305	1471.2376	TRUE
Rm	MS0549	MS0549	1666.6835	TRUE
Rm	BE10	BE11	1297.9527	FALSE
Rm	BE10	BE11	1142.8309	FALSE
Rm	BE10	CH15	2037.9286	FALSE
Rm	BE10	CH15	1610.2285	FALSE
Rm	BE10	CH15	1714.6108	FALSE
Rm	BE10	CH15	2038.7547	FALSE
Rm	BE10	CH15	1643.685	FALSE
Rm	BE10	CH29	1605.8198	FALSE
Rm	BE10	CH29	1382.1076	FALSE
Rm	BE10	CH43	1466.2846	FALSE
Rm	BE10	MS05156	1497.5318	FALSE
Rm	BE10	MS05156	1660.0208	FALSE
Rm	BE10	MS05156	1377.5559	FALSE
Rm	BE10	MS05298	1656.2516	FALSE
Rm	BE10	MS05298	1490.2784	FALSE
Rm	BE10	MS05298	1350.8232	FALSE

Rm	BE10	MS05298	1495.2759	FALSE
Rm	BE10	MS05305	1386.4879	FALSE
Rm	BE10	MS05305	1486.0983	FALSE
Rm	BE10	MS05305	1750.2827	FALSE
Rm	BE10	MS05305	1488.8021	FALSE
Rm	BE10	MS05375	1731.2982	FALSE
Rm	BE10	MS0549	1615.9299	FALSE
Rm	BE10	MS0549	1742.7406	FALSE
Rm	BE10	MS05591	2153.4841	FALSE
Rm	BE11	CH15	2292.1643	FALSE
Rm	BE11	CH15	1789.2092	FALSE
Rm	BE11	CH15	1790.6798	FALSE
Rm	BE11	CH15	2230.5213	FALSE
Rm	BE11	CH15	1884.4042	FALSE
Rm	BE11	CH15	1981.5706	FALSE
Rm	BE11	CH15	1583.5329	FALSE
Rm	BE11	CH15	1514.9932	FALSE
Rm	BE11	CH15	1919.8627	FALSE
Rm	BE11	CH15	1580.3385	FALSE
Rm	BE11	CH29	1760.8985	FALSE
Rm	BE11	CH29	1624.0146	FALSE
Rm	BE11	CH29	1545.0684	FALSE
Rm	BE11	CH29	1347.0235	FALSE
Rm	BE11	CH43	1656.0838	FALSE
Rm	BE11	CH43	1412.746	FALSE
Rm	BE11	MS05156	1626.612	FALSE
Rm	BE11	MS05156	1807.4103	FALSE
Rm	BE11	MS05156	1638.4607	FALSE
Rm	BE11	MS05156	1443.1188	FALSE
Rm	BE11	MS05156	1669.9293	FALSE
Rm	BE11	MS05156	1392.5897	FALSE
Rm	BE11	MS05298	1793.7324	FALSE
Rm	BE11	MS05298	1665.1113	FALSE
Rm	BE11	MS05298	1634.4609	FALSE
Rm	BE11	MS05298	1744.5334	FALSE
Rm	BE11	MS05298	1563.3517	FALSE
Rm	BE11	MS05298	1476.4432	FALSE
Rm	BE11	MS05298	1366.0748	FALSE
Rm	BE11	MS05298	1634.1592	FALSE
Rm	BE11	MS05305	1478.952	FALSE
Rm	BE11	MS05305	1639.1412	FALSE
Rm	BE11	MS05305	1905.5171	FALSE
Rm	BE11	MS05305	1684.4127	FALSE
Rm	BE11	MS05305	1286.5622	FALSE
Rm	BE11	MS05305	1506.2628	FALSE
Rm	BE11	MS05305	1616.7108	FALSE
Rm	BE11	MS05305	1463.9733	FALSE
Rm	BE11	MS05375	1918.3977	FALSE
Rm	BE11	MS05375	1693.5525	FALSE
Rm	BE11	MS0549	1725.1531	FALSE

Rm	BE11	MS0549	1943.5356	FALSE
Rm	BE11	MS0549	1501.3424	FALSE
Rm	BE11	MS0549	1759.4048	FALSE
Rm	BE11	MS05591	2341.4299	FALSE
Rm	BE11	MS05591	2099.8365	FALSE
Rm	CH15	CH29	1896.1606	FALSE
Rm	CH15	CH29	1791.4806	FALSE
Rm	CH15	CH29	1312.9899	FALSE
Rm	CH15	CH29	1277.8983	FALSE
Rm	CH15	CH29	1336.3715	FALSE
Rm	CH15	CH29	1174.6994	FALSE
Rm	CH15	CH29	1673.7193	FALSE
Rm	CH15	CH29	1554.7066	FALSE
Rm	CH15	CH29	1227.2939	FALSE
Rm	CH15	CH29	1167.3333	FALSE
Rm	CH15	CH43	1716.7339	FALSE
Rm	CH15	CH43	1258.4404	FALSE
Rm	CH15	CH43	1347.3698	FALSE
Rm	CH15	CH43	1737.0416	FALSE
Rm	CH15	CH43	1379.3826	FALSE
Rm	CH15	MS05156	1912.154	FALSE
Rm	CH15	MS05156	2104.5038	FALSE
Rm	CH15	MS05156	1828.5892	FALSE
Rm	CH15	MS05156	1468.2726	FALSE
Rm	CH15	MS05156	1655.4909	FALSE
Rm	CH15	MS05156	1280.0607	FALSE
Rm	CH15	MS05156	1509.3366	FALSE
Rm	CH15	MS05156	1570.3323	FALSE
Rm	CH15	MS05156	1275.2303	FALSE
Rm	CH15	MS05156	1824.7861	FALSE
Rm	CH15	MS05156	1970.9141	FALSE
Rm	CH15	MS05156	1743.3704	FALSE
Rm	CH15	MS05156	1610.5951	FALSE
Rm	CH15	MS05156	1779.0709	FALSE
Rm	CH15	MS05156	1345.6263	FALSE
Rm	CH15	MS05298	1952.8659	FALSE
Rm	CH15	MS05298	1829.5409	FALSE
Rm	CH15	MS05298	1634.4093	FALSE
Rm	CH15	MS05298	1851.3346	FALSE
Rm	CH15	MS05298	1610.3038	FALSE
Rm	CH15	MS05298	1244.8223	FALSE
Rm	CH15	MS05298	1265.2575	FALSE
Rm	CH15	MS05298	1359.414	FALSE
Rm	CH15	MS05298	1518.3209	FALSE
Rm	CH15	MS05298	1334.3332	FALSE
Rm	CH15	MS05298	1292.5087	FALSE
Rm	CH15	MS05298	1373.7842	FALSE
Rm	CH15	MS05298	1934.9894	FALSE
Rm	CH15	MS05298	1621.2339	FALSE
Rm	CH15	MS05298	1573.2184	FALSE

Rm	CH15	MS05298	1720.0088	FALSE
Rm	CH15	MS05298	1698.0759	FALSE
Rm	CH15	MS05298	1135.3391	FALSE
Rm	CH15	MS05298	1252.748	FALSE
Rm	CH15	MS05298	1285.5443	FALSE
Rm	CH15	MS05305	1561.7027	FALSE
Rm	CH15	MS05305	1830.5909	FALSE
Rm	CH15	MS05305	2015.0797	FALSE
Rm	CH15	MS05305	2018.2245	FALSE
Rm	CH15	MS05305	1204.6979	FALSE
Rm	CH15	MS05305	1311.888	FALSE
Rm	CH15	MS05305	1561.3568	FALSE
Rm	CH15	MS05305	1375.1696	FALSE
Rm	CH15	MS05305	1225.577	FALSE
Rm	CH15	MS05305	1359.4078	FALSE
Rm	CH15	MS05305	1656.2099	FALSE
Rm	CH15	MS05305	1376.1814	FALSE
Rm	CH15	MS05305	1594.7055	FALSE
Rm	CH15	MS05305	1575.2436	FALSE
Rm	CH15	MS05305	2008.6361	FALSE
Rm	CH15	MS05305	1616.3077	FALSE
Rm	CH15	MS05305	1199.2228	FALSE
Rm	CH15	MS05305	1333.5272	FALSE
Rm	CH15	MS05305	1637.3319	FALSE
Rm	CH15	MS05305	1373.2804	FALSE
Rm	CH15	MS05375	2184.2679	FALSE
Rm	CH15	MS05375	1675.137	FALSE
Rm	CH15	MS05375	1775.0564	FALSE
Rm	CH15	MS05375	2044.3217	FALSE
Rm	CH15	MS05375	1915.4896	FALSE
Rm	CH15	MS0549	1863.4226	FALSE
Rm	CH15	MS0549	2195.8486	FALSE
Rm	CH15	MS0549	1429.1399	FALSE
Rm	CH15	MS0549	1757.5827	FALSE
Rm	CH15	MS0549	1458.2397	FALSE
Rm	CH15	MS0549	1724.7186	FALSE
Rm	CH15	MS0549	1854.2382	FALSE
Rm	CH15	MS0549	2094.9861	FALSE
Rm	CH15	MS0549	1457.9665	FALSE
Rm	CH15	MS0549	1837.7736	FALSE
Rm	CH15	MS05591	2394.7286	FALSE
Rm	CH15	MS05591	1991.3333	FALSE
Rm	CH15	MS05591	1852.9314	FALSE
Rm	CH15	MS05591	2229.5176	FALSE
Rm	CH15	MS05591	2155.0769	FALSE
Rm	CH29	CH43	1238.8027	FALSE
Rm	CH29	CH43	1154.0682	FALSE
Rm	CH29	MS05156	1402.9684	FALSE
Rm	CH29	MS05156	1562.1268	FALSE
Rm	CH29	MS05156	1165.429	FALSE

Rm	CH29	MS05156	1307.0246	FALSE
Rm	CH29	MS05156	1410.2013	FALSE
Rm	CH29	MS05156	1164.1264	FALSE
Rm	CH29	MS05298	1530.2857	FALSE
Rm	CH29	MS05298	1063.7693	FALSE
Rm	CH29	MS05298	1223.0153	FALSE
Rm	CH29	MS05298	1070.7501	FALSE
Rm	CH29	MS05298	1333.4221	FALSE
Rm	CH29	MS05298	1140.1194	FALSE
Rm	CH29	MS05298	1124.1403	FALSE
Rm	CH29	MS05298	1160.7568	FALSE
Rm	CH29	MS05305	1061.6695	FALSE
Rm	CH29	MS05305	1209.3558	FALSE
Rm	CH29	MS05305	1500.6873	FALSE
Rm	CH29	MS05305	1262.4369	FALSE
Rm	CH29	MS05305	994.1343	FALSE
Rm	CH29	MS05305	1128.7952	FALSE
Rm	CH29	MS05305	1516.6878	FALSE
Rm	CH29	MS05305	1212.826	FALSE
Rm	CH29	MS05375	1726.3528	FALSE
Rm	CH29	MS05375	1490.1128	FALSE
Rm	CH29	MS0549	1305.6201	FALSE
Rm	CH29	MS0549	1704.6772	FALSE
Rm	CH29	MS0549	1242.9438	FALSE
Rm	CH29	MS0549	1528.2144	FALSE
Rm	CH29	MS05591	1860.826	FALSE
Rm	CH29	MS05591	1747.4674	FALSE
Rm	CH43	MS05156	1365.392	FALSE
Rm	CH43	MS05156	1464.4965	FALSE
Rm	CH43	MS05156	1218.6447	FALSE
Rm	CH43	MS05298	1450.7276	FALSE
Rm	CH43	MS05298	1185.4241	FALSE
Rm	CH43	MS05298	1202.105	FALSE
Rm	CH43	MS05298	1120.7715	FALSE
Rm	CH43	MS05305	1062.5787	FALSE
Rm	CH43	MS05305	1221.4536	FALSE
Rm	CH43	MS05305	1485.5345	FALSE
Rm	CH43	MS05305	1221.8904	FALSE
Rm	CH43	MS05375	1516.7898	FALSE
Rm	CH43	MS0549	1327.2684	FALSE
Rm	CH43	MS0549	1545.945	FALSE
Rm	CH43	MS05591	1909.3225	FALSE
Rm	MS05156	MS05298	1422.654	FALSE
Rm	MS05156	MS05298	1345.519	FALSE
Rm	MS05156	MS05298	1304.1302	FALSE
Rm	MS05156	MS05298	1354.0667	FALSE
Rm	MS05156	MS05298	1587.1133	FALSE
Rm	MS05156	MS05298	1441.6707	FALSE
Rm	MS05156	MS05298	1441.2328	FALSE
Rm	MS05156	MS05298	1442.7594	FALSE

Rm	MS05156	MS05298	1347.6837	FALSE
Rm	MS05156	MS05298	1086.5889	FALSE
Rm	MS05156	MS05298	1178.7318	FALSE
Rm	MS05156	MS05298	1103.3422	FALSE
Rm	MS05156	MS05305	1231.1618	FALSE
Rm	MS05156	MS05305	1279.0308	FALSE
Rm	MS05156	MS05305	1588.8366	FALSE
Rm	MS05156	MS05305	1372.0235	FALSE
Rm	MS05156	MS05305	1438.3461	FALSE
Rm	MS05156	MS05305	1520.0586	FALSE
Rm	MS05156	MS05305	1762.6647	FALSE
Rm	MS05156	MS05305	1512.731	FALSE
Rm	MS05156	MS05305	1082.1631	FALSE
Rm	MS05156	MS05305	1140.5323	FALSE
Rm	MS05156	MS05305	1528.6766	FALSE
Rm	MS05156	MS05305	1244.5543	FALSE
Rm	MS05156	MS05375	1608.2467	FALSE
Rm	MS05156	MS05375	1811.8544	FALSE
Rm	MS05156	MS05375	1547.6898	FALSE
Rm	MS05156	MS0549	1450.6325	FALSE
Rm	MS05156	MS0549	1569.0891	FALSE
Rm	MS05156	MS0549	1597.1654	FALSE
Rm	MS05156	MS0549	1691.0026	FALSE
Rm	MS05156	MS0549	1356.3302	FALSE
Rm	MS05156	MS0549	1533.9525	FALSE
Rm	MS05156	MS05591	2083.3066	FALSE
Rm	MS05156	MS05591	2056.8369	FALSE
Rm	MS05156	MS05591	2024.825	FALSE
Rm	MS05298	MS05305	1296.945	FALSE
Rm	MS05298	MS05305	1397.7184	FALSE
Rm	MS05298	MS05305	1739.8021	FALSE
Rm	MS05298	MS05305	1425.7697	FALSE
Rm	MS05298	MS05305	1016.2667	FALSE
Rm	MS05298	MS05305	1115.4916	FALSE
Rm	MS05298	MS05305	1463.0959	FALSE
Rm	MS05298	MS05305	1154.7053	FALSE
Rm	MS05298	MS05305	1061.3204	FALSE
Rm	MS05298	MS05305	1135.4447	FALSE
Rm	MS05298	MS05305	1423.6318	FALSE
Rm	MS05298	MS05305	1242.1924	FALSE
Rm	MS05298	MS05305	1083.3139	FALSE
Rm	MS05298	MS05305	1263.321	FALSE
Rm	MS05298	MS05305	1518.5368	FALSE
Rm	MS05298	MS05305	1235.5951	FALSE
Rm	MS05298	MS05375	1651.5172	FALSE
Rm	MS05298	MS05375	1612.813	FALSE
Rm	MS05298	MS05375	1509.2795	FALSE
Rm	MS05298	MS05375	1664.4321	FALSE
Rm	MS05298	MS0549	1498.9582	FALSE
Rm	MS05298	MS0549	1641.5103	FALSE

Rm	MS05298	MS0549	1280.4615	FALSE
Rm	MS05298	MS0549	1607.7457	FALSE
Rm	MS05298	MS0549	1269.5111	FALSE
Rm	MS05298	MS0549	1416.1093	FALSE
Rm	MS05298	MS0549	1306.9939	FALSE
Rm	MS05298	MS0549	1708.9952	FALSE
Rm	MS05298	MS05591	2019.3564	FALSE
Rm	MS05298	MS05591	1891.7393	FALSE
Rm	MS05298	MS05591	1776.1623	FALSE
Rm	MS05298	MS05591	1997.032	FALSE
Rm	MS05305	MS05375	1388.9162	FALSE
Rm	MS05305	MS05375	1567.1752	FALSE
Rm	MS05305	MS05375	1820.2884	FALSE
Rm	MS05305	MS05375	1591.026	FALSE
Rm	MS05305	MS0549	1109.7629	FALSE
Rm	MS05305	MS0549	1403.1783	FALSE
Rm	MS05305	MS0549	1307.1532	FALSE
Rm	MS05305	MS0549	1584.4069	FALSE
Rm	MS05305	MS0549	1641.0562	FALSE
Rm	MS05305	MS0549	1868.2417	FALSE
Rm	MS05305	MS0549	1318.4577	FALSE
Rm	MS05305	MS0549	1600.9143	FALSE
Rm	MS05305	MS05591	1752.2735	FALSE
Rm	MS05305	MS05591	1886.882	FALSE
Rm	MS05305	MS05591	1947.0755	FALSE
Rm	MS05305	MS05591	1836.2923	FALSE
Rm	MS05375	MS0549	1702.021	FALSE
Rm	MS05375	MS0549	1963.9256	FALSE
Rm	MS05375	MS05591	2303.1356	FALSE
Rm	MS0549	MS05591	1941.9816	FALSE
Rm	MS0549	MS05591	2339.1478	FALSE
Cg	CH50	CH50	1877.3006	TRUE
Cg	CH50	CH50	843.4955	TRUE
Cg	CH50	CH50	1785.8631	TRUE
Cg	DEL9	DEL9	1395.17	TRUE
Cg	DEL9	DEL9	1735.897	TRUE
Cg	DEL9	DEL9	1529.8155	TRUE
Cg	DEL9	DEL9	1534.9978	TRUE
Cg	DEL9	DEL9	1375.4088	TRUE
Cg	DEL9	DEL9	1613.2581	TRUE
Cg	MS0521	MS0521	1679.5205	TRUE
Cg	MS0521	MS0521	1214.0872	TRUE
Cg	MS0521	MS0521	1073.298	TRUE
Cg	MS0521	MS0521	1368.6528	TRUE
Cg	MS0521	MS0521	1572.0225	TRUE
Cg	MS0521	MS0521	887.5932	TRUE
Cg	MS05403	MS05403	1910.5415	TRUE
Cg	MS05403	MS05403	2374.9116	TRUE
Cg	MS05403	MS05403	2355.3364	TRUE
Cg	MS05489	MS05489	1949.7959	TRUE

Cg	MS05489	MS05489	1745.8192	TRUE
Cg	MS05489	MS05489	2075.8116	TRUE
Cg	MS05489	MS05489	1424.8492	TRUE
Cg	MS05489	MS05489	1893.5552	TRUE
Cg	MS05489	MS05489	1731.4246	TRUE
Cg	MS055	MS055	1060.9844	TRUE
Cg	MS055	MS055	1489.9065	TRUE
Cg	MS055	MS055	1125.2973	TRUE
Cg	MS055	MS055	1017.3623	TRUE
Cg	MS055	MS055	818.7859	TRUE
Cg	MS055	MS055	1001.2002	TRUE
Cg	MS0583	MS0583	1056.5092	TRUE
Cg	MS0583	MS0583	1095.7347	TRUE
Cg	MS0583	MS0583	1423.6951	TRUE
Cg	AU3	CH50	1617.9536	FALSE
Cg	AU3	CH50	1739.3321	FALSE
Cg	AU3	CH50	1423.9775	FALSE
Cg	AU3	DEL5	1405.3386	FALSE
Cg	AU3	DEL9	1570.5549	FALSE
Cg	AU3	DEL9	1326.648	FALSE
Cg	AU3	DEL9	1716.4875	FALSE
Cg	AU3	DEL9	1511.6921	FALSE
Cg	AU3	MS0521	1474.731	FALSE
Cg	AU3	MS0521	1610.2164	FALSE
Cg	AU3	MS0521	1054.3619	FALSE
Cg	AU3	MS0521	1170.684	FALSE
Cg	AU3	MS05396	1652.8799	FALSE
Cg	AU3	MS05403	1732.7647	FALSE
Cg	AU3	MS05403	1839.7255	FALSE
Cg	AU3	MS05403	1883.2392	FALSE
Cg	AU3	MS05457	1042.4543	FALSE
Cg	AU3	MS05488	1633.3182	FALSE
Cg	AU3	MS05489	1964.9293	FALSE
Cg	AU3	MS05489	1733.709	FALSE
Cg	AU3	MS05489	1516.1934	FALSE
Cg	AU3	MS05489	1790.3293	FALSE
Cg	AU3	MS055	1507.4008	FALSE
Cg	AU3	MS055	1188.8796	FALSE
Cg	AU3	MS055	1557.7261	FALSE
Cg	AU3	MS055	1192.2491	FALSE
Cg	AU3	MS0583	1182.3927	FALSE
Cg	AU3	MS0583	1582.999	FALSE
Cg	AU3	MS0583	1536.9477	FALSE
Cg	AU3	VJ6	1680.4329	FALSE
Cg	CH50	DEL5	1823.6134	FALSE
Cg	CH50	DEL5	1939.6763	FALSE
Cg	CH50	DEL5	1450.1596	FALSE
Cg	CH50	DEL9	1455.3267	FALSE
Cg	CH50	DEL9	1461.5007	FALSE
Cg	CH50	DEL9	1505.4109	FALSE

Cg	CH50	DEL9	1534.9241	FALSE
Cg	CH50	DEL9	1952.8793	FALSE
Cg	CH50	DEL9	1919.0956	FALSE
Cg	CH50	DEL9	2348.2616	FALSE
Cg	CH50	DEL9	1909.6677	FALSE
Cg	CH50	DEL9	1418.2159	FALSE
Cg	CH50	DEL9	1201.0099	FALSE
Cg	CH50	DEL9	1385.3584	FALSE
Cg	CH50	DEL9	1439.3068	FALSE
Cg	CH50	MS0521	1440.512	FALSE
Cg	CH50	MS0521	1894.6057	FALSE
Cg	CH50	MS0521	986.2221	FALSE
Cg	CH50	MS0521	1151.1339	FALSE
Cg	CH50	MS0521	1970.6133	FALSE
Cg	CH50	MS0521	2146.8661	FALSE
Cg	CH50	MS0521	1602.2277	FALSE
Cg	CH50	MS0521	1712.5899	FALSE
Cg	CH50	MS0521	1249.302	FALSE
Cg	CH50	MS0521	1625.8349	FALSE
Cg	CH50	MS0521	1045.7715	FALSE
Cg	CH50	MS0521	1140.5076	FALSE
Cg	CH50	MS05396	2028.8265	FALSE
Cg	CH50	MS05396	2101.6105	FALSE
Cg	CH50	MS05396	1629.73	FALSE
Cg	CH50	MS05403	1890.4397	FALSE
Cg	CH50	MS05403	1876.6257	FALSE
Cg	CH50	MS05403	1690.0582	FALSE
Cg	CH50	MS05403	2089.8661	FALSE
Cg	CH50	MS05403	2235.3857	FALSE
Cg	CH50	MS05403	2357.7315	FALSE
Cg	CH50	MS05403	1748.9719	FALSE
Cg	CH50	MS05403	1803.2684	FALSE
Cg	CH50	MS05403	1541.9467	FALSE
Cg	CH50	MS05457	1950.0307	FALSE
Cg	CH50	MS05457	2015.1166	FALSE
Cg	CH50	MS05457	1628.2598	FALSE
Cg	CH50	MS05488	1684.6625	FALSE
Cg	CH50	MS05488	1915.0532	FALSE
Cg	CH50	MS05488	1521.8408	FALSE
Cg	CH50	MS05489	1852.9195	FALSE
Cg	CH50	MS05489	1433.456	FALSE
Cg	CH50	MS05489	1444.3474	FALSE
Cg	CH50	MS05489	1781.8915	FALSE
Cg	CH50	MS05489	2276.5363	FALSE
Cg	CH50	MS05489	1959.2436	FALSE
Cg	CH50	MS05489	2019.4739	FALSE
Cg	CH50	MS05489	2090.6828	FALSE
Cg	CH50	MS05489	1754.9514	FALSE
Cg	CH50	MS05489	1251.7054	FALSE
Cg	CH50	MS05489	1318.9904	FALSE

Cg	CH50	MS05489	1592.9761	FALSE
Cg	CH50	MS055	1531.1275	FALSE
Cg	CH50	MS055	1127.314	FALSE
Cg	CH50	MS055	1369.098	FALSE
Cg	CH50	MS055	1153.1322	FALSE
Cg	CH50	MS055	1983.6364	FALSE
Cg	CH50	MS055	1856.0942	FALSE
Cg	CH50	MS055	2041.74	FALSE
Cg	CH50	MS055	1721.1908	FALSE
Cg	CH50	MS055	1394.8072	FALSE
Cg	CH50	MS055	1050.9446	FALSE
Cg	CH50	MS055	1164.9024	FALSE
Cg	CH50	MS055	1050.1604	FALSE
Cg	CH50	MS0583	1245.2545	FALSE
Cg	CH50	MS0583	1430.3994	FALSE
Cg	CH50	MS0583	1347.7059	FALSE
Cg	CH50	MS0583	1777.4208	FALSE
Cg	CH50	MS0583	1951.789	FALSE
Cg	CH50	MS0583	2032.1578	FALSE
Cg	CH50	MS0583	1082.552	FALSE
Cg	CH50	MS0583	1351.4659	FALSE
Cg	CH50	MS0583	1225.1555	FALSE
Cg	CH50	VJ6	1439.6211	FALSE
Cg	CH50	VJ6	2042.4459	FALSE
Cg	CH50	VJ6	1432.0311	FALSE
Cg	DEL5	DEL9	1728.4241	FALSE
Cg	DEL5	DEL9	1453.5754	FALSE
Cg	DEL5	DEL9	1761.6918	FALSE
Cg	DEL5	DEL9	1792.9821	FALSE
Cg	DEL5	MS0521	1738.9835	FALSE
Cg	DEL5	MS0521	1978.3676	FALSE
Cg	DEL5	MS0521	1351.9628	FALSE
Cg	DEL5	MS0521	1329.9457	FALSE
Cg	DEL5	MS05396	1512.66	FALSE
Cg	DEL5	MS05403	1613.3576	FALSE
Cg	DEL5	MS05403	1636.5	FALSE
Cg	DEL5	MS05403	2130.9196	FALSE
Cg	DEL5	MS05457	1538.8956	FALSE
Cg	DEL5	MS05488	1858.9536	FALSE
Cg	DEL5	MS05489	2120.9288	FALSE
Cg	DEL5	MS05489	1858.4109	FALSE
Cg	DEL5	MS05489	1730.614	FALSE
Cg	DEL5	MS05489	2007.9755	FALSE
Cg	DEL5	MS055	1734.0559	FALSE
Cg	DEL5	MS055	1456.445	FALSE
Cg	DEL5	MS055	1600.3868	FALSE
Cg	DEL5	MS055	1463.5692	FALSE
Cg	DEL5	MS0583	1394.5203	FALSE
Cg	DEL5	MS0583	1635.8529	FALSE
Cg	DEL5	MS0583	1843.077	FALSE

Cg	DEL5	VJ6	1808.6465	FALSE
Cg	DEL9	MS0521	1534.4202	FALSE
Cg	DEL9	MS0521	1587.1937	FALSE
Cg	DEL9	MS0521	1204.0141	FALSE
Cg	DEL9	MS0521	1356.1142	FALSE
Cg	DEL9	MS0521	1308.364	FALSE
Cg	DEL9	MS0521	1548.6653	FALSE
Cg	DEL9	MS0521	1074.8609	FALSE
Cg	DEL9	MS0521	1157.1028	FALSE
Cg	DEL9	MS0521	1575.574	FALSE
Cg	DEL9	MS0521	1970.7037	FALSE
Cg	DEL9	MS0521	1376.1477	FALSE
Cg	DEL9	MS0521	1451.3978	FALSE
Cg	DEL9	MS0521	1270.5842	FALSE
Cg	DEL9	MS0521	1830.2082	FALSE
Cg	DEL9	MS0521	1333.8412	FALSE
Cg	DEL9	MS0521	1365.178	FALSE
Cg	DEL9	MS05396	1913.2967	FALSE
Cg	DEL9	MS05396	1525.4902	FALSE
Cg	DEL9	MS05396	1864.7878	FALSE
Cg	DEL9	MS05396	1798.8707	FALSE
Cg	DEL9	MS05403	1928.2382	FALSE
Cg	DEL9	MS05403	1997.2663	FALSE
Cg	DEL9	MS05403	1832.424	FALSE
Cg	DEL9	MS05403	1851.7092	FALSE
Cg	DEL9	MS05403	1671.6579	FALSE
Cg	DEL9	MS05403	1734.8457	FALSE
Cg	DEL9	MS05403	2090.351	FALSE
Cg	DEL9	MS05403	1977.8733	FALSE
Cg	DEL9	MS05403	1904.9642	FALSE
Cg	DEL9	MS05403	1889.6544	FALSE
Cg	DEL9	MS05403	2099.1754	FALSE
Cg	DEL9	MS05403	1949.1925	FALSE
Cg	DEL9	MS05457	1941.7202	FALSE
Cg	DEL9	MS05457	1544.2011	FALSE
Cg	DEL9	MS05457	1791.9733	FALSE
Cg	DEL9	MS05457	1840.5871	FALSE
Cg	DEL9	MS05488	1713.7744	FALSE
Cg	DEL9	MS05488	1487.8817	FALSE
Cg	DEL9	MS05488	2013.4198	FALSE
Cg	DEL9	MS05488	1682.0327	FALSE
Cg	DEL9	MS05489	1773.0028	FALSE
Cg	DEL9	MS05489	1617.1419	FALSE
Cg	DEL9	MS05489	1484.1894	FALSE
Cg	DEL9	MS05489	1772.875	FALSE
Cg	DEL9	MS05489	1761.8005	FALSE
Cg	DEL9	MS05489	1480.9558	FALSE
Cg	DEL9	MS05489	1334.1321	FALSE
Cg	DEL9	MS05489	1704.1007	FALSE
Cg	DEL9	MS05489	1954.3504	FALSE

Cg	DEL9	MS05489	1480.6927	FALSE
Cg	DEL9	MS05489	1634.6254	FALSE
Cg	DEL9	MS05489	1817.4298	FALSE
Cg	DEL9	MS05489	1999.6287	FALSE
Cg	DEL9	MS05489	1574.7673	FALSE
Cg	DEL9	MS05489	1556.933	FALSE
Cg	DEL9	MS05489	1862.3831	FALSE
Cg	DEL9	MS055	1448.9844	FALSE
Cg	DEL9	MS055	1271.6115	FALSE
Cg	DEL9	MS055	1460.926	FALSE
Cg	DEL9	MS055	1242.1707	FALSE
Cg	DEL9	MS055	1259.3987	FALSE
Cg	DEL9	MS055	1056.9472	FALSE
Cg	DEL9	MS055	1338.1942	FALSE
Cg	DEL9	MS055	1063.3631	FALSE
Cg	DEL9	MS055	1638.254	FALSE
Cg	DEL9	MS055	1245.7586	FALSE
Cg	DEL9	MS055	1506.9928	FALSE
Cg	DEL9	MS055	1441.2519	FALSE
Cg	DEL9	MS055	1545.311	FALSE
Cg	DEL9	MS055	1162.0118	FALSE
Cg	DEL9	MS055	1532.7782	FALSE
Cg	DEL9	MS055	1250.3116	FALSE
Cg	DEL9	MS0583	1197.7479	FALSE
Cg	DEL9	MS0583	1572.4883	FALSE
Cg	DEL9	MS0583	1573.0245	FALSE
Cg	DEL9	MS0583	1082.7169	FALSE
Cg	DEL9	MS0583	1341.6637	FALSE
Cg	DEL9	MS0583	1400.0448	FALSE
Cg	DEL9	MS0583	1393.9187	FALSE
Cg	DEL9	MS0583	1542.6405	FALSE
Cg	DEL9	MS0583	1600.5893	FALSE
Cg	DEL9	MS0583	1263.2632	FALSE
Cg	DEL9	MS0583	1557.3817	FALSE
Cg	DEL9	MS0583	1549.4034	FALSE
Cg	DEL9	VJ6	1542.482	FALSE
Cg	DEL9	VJ6	1401.6757	FALSE
Cg	DEL9	VJ6	1638.6561	FALSE
Cg	DEL9	VJ6	1553.9466	FALSE
Cg	MS0521	MS05396	1639.5897	FALSE
Cg	MS0521	MS05396	1821.6142	FALSE
Cg	MS0521	MS05396	1401.1185	FALSE
Cg	MS0521	MS05396	1297.8833	FALSE
Cg	MS0521	MS05403	1791.8212	FALSE
Cg	MS0521	MS05403	1755.2509	FALSE
Cg	MS0521	MS05403	1841.2139	FALSE
Cg	MS0521	MS05403	2185.2519	FALSE
Cg	MS0521	MS05403	2097.0719	FALSE
Cg	MS0521	MS05403	2185.4773	FALSE
Cg	MS0521	MS05403	1418.3579	FALSE

Cg	MS0521	MS05403	1499.7574	FALSE
Cg	MS0521	MS05403	1421.3732	FALSE
Cg	MS0521	MS05403	1585.1474	FALSE
Cg	MS0521	MS05403	1571.5056	FALSE
Cg	MS0521	MS05403	1565.2554	FALSE
Cg	MS0521	MS05457	1711.7869	FALSE
Cg	MS0521	MS05457	1962.0482	FALSE
Cg	MS0521	MS05457	1323.0815	FALSE
Cg	MS0521	MS05457	1175.0451	FALSE
Cg	MS0521	MS05488	1551.1052	FALSE
Cg	MS0521	MS05488	1823.0129	FALSE
Cg	MS0521	MS05488	1207.3833	FALSE
Cg	MS0521	MS05488	1507.3338	FALSE
Cg	MS0521	MS05489	1654.0429	FALSE
Cg	MS0521	MS05489	1458.5121	FALSE
Cg	MS0521	MS05489	1438.5819	FALSE
Cg	MS0521	MS05489	1722.3805	FALSE
Cg	MS0521	MS05489	2159.9038	FALSE
Cg	MS0521	MS05489	1709.8174	FALSE
Cg	MS0521	MS05489	1641.4893	FALSE
Cg	MS0521	MS05489	1925.9048	FALSE
Cg	MS0521	MS05489	1621.1865	FALSE
Cg	MS0521	MS05489	1114.1307	FALSE
Cg	MS0521	MS05489	1132.6233	FALSE
Cg	MS0521	MS05489	1408.9703	FALSE
Cg	MS0521	MS05489	1516.3298	FALSE
Cg	MS0521	MS05489	1169.9961	FALSE
Cg	MS0521	MS05489	1135.8891	FALSE
Cg	MS0521	MS05489	1448.6709	FALSE
Cg	MS0521	MS055	1444.8944	FALSE
Cg	MS0521	MS055	1049.5696	FALSE
Cg	MS0521	MS055	1104.0719	FALSE
Cg	MS0521	MS055	1050.041	FALSE
Cg	MS0521	MS055	1630.774	FALSE
Cg	MS0521	MS055	1471.0987	FALSE
Cg	MS0521	MS055	1493.7627	FALSE
Cg	MS0521	MS055	1225.8241	FALSE
Cg	MS0521	MS055	1025.1222	FALSE
Cg	MS0521	MS055	916.7373	FALSE
Cg	MS0521	MS055	960.9255	FALSE
Cg	MS0521	MS055	763.1797	FALSE
Cg	MS0521	MS055	1135.7865	FALSE
Cg	MS0521	MS055	778.0322	FALSE
Cg	MS0521	MS055	1169.6951	FALSE
Cg	MS0521	MS055	982.2883	FALSE
Cg	MS0521	MS0583	1181.9948	FALSE
Cg	MS0521	MS0583	1329.1151	FALSE
Cg	MS0521	MS0583	1440.2163	FALSE
Cg	MS0521	MS0583	1444.3352	FALSE
Cg	MS0521	MS0583	1671.4216	FALSE

Cg	MS0521	MS0583	1836.2668	FALSE
Cg	MS0521	MS0583	851.8767	FALSE
Cg	MS0521	MS0583	1080.8037	FALSE
Cg	MS0521	MS0583	1019.1149	FALSE
Cg	MS0521	MS0583	954.625	FALSE
Cg	MS0521	MS0583	1105.413	FALSE
Cg	MS0521	MS0583	1226.5927	FALSE
Cg	MS05396	MS05403	1822.0297	FALSE
Cg	MS05396	MS05403	1764.8913	FALSE
Cg	MS05396	MS05403	2273.4611	FALSE
Cg	MS05396	MS05457	1709.5651	FALSE
Cg	MS05396	MS05488	1852.9593	FALSE
Cg	MS05396	MS05489	2318.4372	FALSE
Cg	MS05396	MS05489	1947.4278	FALSE
Cg	MS05396	MS05489	1808.4889	FALSE
Cg	MS05396	MS05489	2136.561	FALSE
Cg	MS05396	MS055	1811.3524	FALSE
Cg	MS05396	MS055	1490.9039	FALSE
Cg	MS05396	MS055	1737.2923	FALSE
Cg	MS05396	MS055	1504.0214	FALSE
Cg	MS05396	MS0583	1416.1018	FALSE
Cg	MS05396	MS0583	1714.0711	FALSE
Cg	MS05396	MS0583	1770.1063	FALSE
Cg	MS05403	MS05457	1908.2435	FALSE
Cg	MS05403	MS05457	2005.9209	FALSE
Cg	MS05403	MS05457	2418.4548	FALSE
Cg	MS05403	MS05488	2028.0331	FALSE
Cg	MS05403	MS05488	2073.6225	FALSE
Cg	MS05403	MS05488	1761.027	FALSE
Cg	MS05403	MS05489	2413.048	FALSE
Cg	MS05403	MS05489	1842.0431	FALSE
Cg	MS05403	MS05489	1933.5964	FALSE
Cg	MS05403	MS05489	2138.7622	FALSE
Cg	MS05403	MS05489	2184.1733	FALSE
Cg	MS05403	MS05489	2051.7787	FALSE
Cg	MS05403	MS05489	2000.007	FALSE
Cg	MS05403	MS05489	2233.5911	FALSE
Cg	MS05403	MS05489	2210.2003	FALSE
Cg	MS05403	MS05489	1832.4728	FALSE
Cg	MS05403	MS05489	1612.468	FALSE
Cg	MS05403	MS05489	2046.6402	FALSE
Cg	MS05403	MS055	1885.3924	FALSE
Cg	MS05403	MS055	1822.2989	FALSE
Cg	MS05403	MS055	1869.1495	FALSE
Cg	MS05403	MS055	1753.2923	FALSE
Cg	MS05403	MS055	1930.2308	FALSE
Cg	MS05403	MS055	1622.4016	FALSE
Cg	MS05403	MS055	1665.4558	FALSE
Cg	MS05403	MS055	1678.6079	FALSE
Cg	MS05403	MS055	1840.0938	FALSE

Cg	MS05403	MS055	1412.8718	FALSE
Cg	MS05403	MS055	1479.3703	FALSE
Cg	MS05403	MS055	1441.6602	FALSE
Cg	MS05403	MS0583	1620.3722	FALSE
Cg	MS05403	MS0583	1991.6669	FALSE
Cg	MS05403	MS0583	1913.4936	FALSE
Cg	MS05403	MS0583	1687.6999	FALSE
Cg	MS05403	MS0583	1869.0955	FALSE
Cg	MS05403	MS0583	1987.123	FALSE
Cg	MS05403	MS0583	1524.0687	FALSE
Cg	MS05403	MS0583	1834.3024	FALSE
Cg	MS05403	MS0583	1799.4848	FALSE
Cg	MS05457	MS05488	1760.6789	FALSE
Cg	MS05457	MS05489	2198.1331	FALSE
Cg	MS05457	MS05489	2029.6789	FALSE
Cg	MS05457	MS05489	1812.7914	FALSE
Cg	MS05457	MS05489	2069.7187	FALSE
Cg	MS05457	MS055	1857.9932	FALSE
Cg	MS05457	MS055	1481.7302	FALSE
Cg	MS05457	MS055	1715.9871	FALSE
Cg	MS05457	MS055	1490.8429	FALSE
Cg	MS05457	MS0583	1453.167	FALSE
Cg	MS05457	MS0583	1887.5587	FALSE
Cg	MS05457	MS0583	1748.3573	FALSE
Cg	MS05488	MS05489	1955.2458	FALSE
Cg	MS05488	MS05489	1696.0642	FALSE
Cg	MS05488	MS05489	1387.4477	FALSE
Cg	MS05488	MS05489	1812.3223	FALSE
Cg	MS05488	MS055	1485.4255	FALSE
Cg	MS05488	MS055	1323.3594	FALSE
Cg	MS05488	MS055	1726.4176	FALSE
Cg	MS05488	MS055	1425.7525	FALSE
Cg	MS05488	MS0583	1340.0501	FALSE
Cg	MS05488	MS0583	1597.1954	FALSE
Cg	MS05488	MS0583	1709.0156	FALSE
Cg	MS05489	MS055	1687.7348	FALSE
Cg	MS05489	MS055	1471.9708	FALSE
Cg	MS05489	MS055	1676.029	FALSE
Cg	MS05489	MS055	1621.8156	FALSE
Cg	MS05489	MS055	1652.3604	FALSE
Cg	MS05489	MS055	1345.9495	FALSE
Cg	MS05489	MS055	1452.7219	FALSE
Cg	MS05489	MS055	1225.4062	FALSE
Cg	MS05489	MS055	1382.2545	FALSE
Cg	MS05489	MS055	1209.5407	FALSE
Cg	MS05489	MS055	1424.9524	FALSE
Cg	MS05489	MS055	1091	FALSE
Cg	MS05489	MS055	1736.8646	FALSE
Cg	MS05489	MS055	1514.8527	FALSE
Cg	MS05489	MS055	1420.2785	FALSE

Cg	MS05489	MS055	1538.6159	FALSE
Cg	MS05489	MS0583	1597.9437	FALSE
Cg	MS05489	MS0583	1931.6587	FALSE
Cg	MS05489	MS0583	1764.6969	FALSE
Cg	MS05489	MS0583	1329.2644	FALSE
Cg	MS05489	MS0583	1579.1192	FALSE
Cg	MS05489	MS0583	1342.5024	FALSE
Cg	MS05489	MS0583	1129.5356	FALSE
Cg	MS05489	MS0583	1401.719	FALSE
Cg	MS05489	MS0583	1334.1967	FALSE
Cg	MS05489	MS0583	1447.8692	FALSE
Cg	MS05489	MS0583	1713.1649	FALSE
Cg	MS05489	MS0583	1681.6618	FALSE
Cg	MS055	MS0583	1179.3966	FALSE
Cg	MS055	MS0583	1473.9171	FALSE
Cg	MS055	MS0583	1436.3013	FALSE
Cg	MS055	MS0583	890.3879	FALSE
Cg	MS055	MS0583	984.5777	FALSE
Cg	MS055	MS0583	1087.1119	FALSE
Cg	MS055	MS0583	1140.2053	FALSE
Cg	MS055	MS0583	1381.7618	FALSE
Cg	MS055	MS0583	1278.6152	FALSE
Cg	MS055	MS0583	964.9936	FALSE
Cg	MS055	MS0583	1121.5462	FALSE
Cg	MS055	MS0583	1196.3807	FALSE
Cg	VJ6	MS0521	1542.6879	FALSE
Cg	VJ6	MS0521	1727.8193	FALSE
Cg	VJ6	MS0521	1225.228	FALSE
Cg	VJ6	MS0521	1105.6247	FALSE
Cg	VJ6	MS05396	1905.0722	FALSE
Cg	VJ6	MS05403	2058.0227	FALSE
Cg	VJ6	MS05403	2077.8929	FALSE
Cg	VJ6	MS05403	1885.679	FALSE
Cg	VJ6	MS05457	2015.3283	FALSE
Cg	VJ6	MS05488	1576.197	FALSE
Cg	VJ6	MS05489	1866.604	FALSE
Cg	VJ6	MS05489	1568.5304	FALSE
Cg	VJ6	MS05489	1404.7482	FALSE
Cg	VJ6	MS05489	1784.6499	FALSE
Cg	VJ6	MS055	1539.0552	FALSE
Cg	VJ6	MS055	1140.5909	FALSE
Cg	VJ6	MS055	1505.3992	FALSE
Cg	VJ6	MS055	1206.4193	FALSE
Cg	VJ6	MS0583	1053.3303	FALSE
Cg	VJ6	MS0583	1723.2669	FALSE
Cg	VJ6	MS0583	1424.6775	FALSE

Abbreviations: Rm–*Rhinoptericola megacantha*; Cg–*Callitetrarhynchus gracilis*

Supplementary Table S7. Statistical tests comparing the distributions of pairwise distances measured within infracommunities and between pairs of infracommunities for *Rhinoptericola megacantha* and *Callitetrarhynchus gracilis* based on "no-Senegal" single nucleotide polymorphism datasets. Pairwise distances calculated for individual worms that were collected from either the same, or from two different, host individuals were combined into two separate distributions. Each distribution was then tested for normality using a Jarque-Bera test (Bera and Jarque 1981). All data were normally distributed excepting the distribution of pairwise distances calculated for *R. megacantha* sampled from between different host individuals. For *C. gracilis*, in which both datasets were found to be normally distributed, an F-tests was performed to determine whether the variances of the two distributions (i.e., within versus between host individuals) differed significantly. The p-value for this test was significant, and so a two-tailed T-test assuming unequal variance (Student 1908) was performed. The p-value for this test was 0.15, suggesting no significant difference between pairwise distances calculated within versus between host individuals for *C. gracilis*. For *R. megacantha*, in which one of the two distributions was determined not to be normally distributed, a non-parametric Kolmogorov-Smirnov test was performed. The p-value for this test was 0.126, demonstrating no significant difference between the means of the two datasets for *R. megacantha*. (For thoroughness, an F-test indicated no significant difference in variance between the two datasets, and a two-tailed T-test assuming equal variance [Student 1908] resulted in a p-value of 0.18, similarly indicating no significant difference between pairwise distances calculated within versus between host individuals for *R. megacantha* based on parametric testing.)

Rhinoptericola megacantha

**Testing for normality of the within-host-individual dataset**	
no. observations	28
skewness	0.713945512
kurtosis	-0.107262775
Jarque-Bera test statistic	2.392107757
p-value	0.302385109
Conclusion	Data are normally distributed

**Testing for normality of the between-host-individual dataset**	
no. observations	297
skewness	0.439011307
kurtosis	-0.261393928
Jarque-Bera test statistic	10.38572487
p-value	0.00555608
Conclusion	Data are NOT normally distributed

**Testing if variances differ between the two datasets: F-Test**	
Variance for within-host-individual dataset	120175.3318
Variance for between-host-individual dataset	85172.09006
Two-sample F-test	0.178395279
Conclusion	Variances do NOT differ significantly; can assume equal variance in T-test

Callitetrarhynchus gracilis

**Testing for normality of the within-host-individual dataset**	
no. observations	33
skewness	0.277187076
kurtosis	-0.541217082
Jarque-Bera test statistic	0.825339116
p-value	0.66188096
Conclusion	Data are normally distributed

**Testing for normality of the between-host-individual dataset**	
no. observations	432
skewness	-0.017816524
kurtosis	-0.454605797
Jarque-Bera test statistic	3.742850611
p-value	0.153904145
Conclusion	Data are normally distributed

**Testing if variances differ between the two datasets: F-Test**	
Variance for within-host-individual dataset	173694.0091
Variance for between-host-individual dataset	102073.2995
Two-sample F-test	0.022260752
Conclusion	Variances DO differ significantly; must assume unequal variance in T-test

Testing if variances differ between the two datasets: F-Test	
Two-sample T-test assuming equal variance	0.201467457
Conclusion	Means of the two distributions are not significantly different

Testing if variances differ between the two datasets: F-Test	
Two-sample T-test assuming unequal variance	0.151914375
Conclusion	Means of the two distributions are not significantly different

Testing if variances differ between the two datasets: Non-parametric Kolmogorov-Smirnov test (since between-host-individual data are not normally distributed)	
alternative hypothesis	two-sided
D	0.22535
p-value	0.126
Conclusion	Means of the two distributions are not significantly different

References

Bera AK, & Jarque CM. 1981. Efficient tests for normality, homoscedasticity and serial independence of regression residuals: Monte Carlo evidence. Econ. Lett. 7(4): 313–318. doi: 10.1016/0165-1765(81)90035-5

Massey Jr FJ. 1951. The Kolmogorov-Smirnov test for goodness of fit. JASA 46(253): 68–78. doi: 10.1080/01621459.1951.10500769

Student. 1908. The probable error of a mean. Biometrika 6(1) 1–25. doi: 10.2307/2331554
